# Fluid Flow and Heat Transfer Analysis of a Nanofluid Containing Motile Gyrotactic Micro-Organisms Passing a Nonlinear Stretching Vertical Sheet in the Presence of a Non-Uniform Magnetic Field; Numerical Approach

**DOI:** 10.1371/journal.pone.0157598

**Published:** 2016-06-20

**Authors:** S. A. M. Mehryan, Farshad Moradi Kashkooli, M. Soltani, Kaamran Raahemifar

**Affiliations:** 1 Department of Mechanical Engineering, K. N. T. University of Technology, Tehran, Iran; 2 Division of Nuclear Medicine, Department of Radiology and Radiological Science, Johns Hopkins University, School of Medicine, Baltimore, MD, United States of America; 3 Electrical & Computer Engineering Department of Ryerson University, Toronto, Ontario, Canada; The Ohio State University, UNITED STATES

## Abstract

The behavior of a water-based nanofluid containing motile gyrotactic micro-organisms passing an isothermal nonlinear stretching sheet in the presence of a non-uniform magnetic field is studied numerically. The governing partial differential equations including continuity, momentums, energy, concentration of the nanoparticles, and density of motile micro-organisms are converted into a system of the ordinary differential equations via a set of similarity transformations. New set of equations are discretized using the finite difference method and have been linearized by employing the Newton’s linearization technique. The tri-diagonal system of algebraic equations from discretization is solved using the well-known Thomas algorithm. The numerical results for profiles of velocity, temperature, nanoparticles concentration and density of motile micro-organisms as well as the local skin friction coefficient *C*_*fx*_, the local Nusselt number *Nu*_*x*_, the local Sherwood number *Sh*_*x*_ and the local density number of the motile microorganism *Nn*_*x*_ are expressed graphically and described in detail. This investigation shows the density number of the motile micro-organisms enhances with rise of *M*, *Gr/Re*^*2*^, *Pe* and *Ω* but it decreases with augment of *Rb* and *n*. Also, Sherwood number augments with an increase of *M* and *Gr/Re*^*2*^, while decreases with *n*, *Rb*, *Nb* and *Nr*. To show the validity of the current results, a comparison between the present results and the existing literature has been carried out.

## Introduction

The problem of boundary layer flow passing a stretching sheet has been an important and interesting challenge for research studies due to its numerous applications in industry and engineering. Some of these applications consist of cooling of papers, glass-fiber production, plastic sheets and polymer extrusion, hot rolling wire drawing, metal spinning, glass blowing, stretching of rubber sheets and plastics, and in textile industry. In these processes, the rate of stretching and cooling has significant effects on the quality and final product formation.

The investigation of the boundary layer flow past a flat plate with constant speed began first by Sakiadis [1]. After Sakiadis, a large number of research papers addressed the boundary layer flow passing the stretching surfaces considering different parameters such as the blowing or suction [2–3], porosity [4–5] and magnetic field [6–7] and various types of fluids such as Newtonian [8], and polar fluids [9], non-Newtonian [7, 10–11]. To improve the thermal properties, researchers presented nanofluids (liquid containing nanometer-sized particles). They have widely used in engineering (such as cooling), biomedical (such as cancer therapy) and process industries. Fluid flow and heat transfer in a formed boundary layer flow due to the nanofluids movement on a stretching sheet includes the wide range of recent researches. A very important issue in the boundary layer flow is the heat transfer characteristics; because, as mentioned, the quality of the final products depends on the rate of heat transfer. Therefore, nanofluids due to the high thermal conductivity of nanoparticles can be used to increase the heat transfer rate [11–12]. Kuznetsov and Nield [13] analytically examined the natural convection of a nanofluid passing a vertical sheet with consideration of the Brownian motion and the thermophoresis effects. Noghrehabadadi et al. [14] carried out the flow and heat transfer of nanofluids over stretching sheet considering partial slip and thermal convective boundary conditions. Zaraki et al. [15] numerically investigated the effects of the shape, size and type of nanoparticles, type of base fluid and working temperature on the flow and heat transfer characteristics of a natural convection boundary layer. Makinde and Aziz [16] conducted a numerical study of nanofluid boundary layer flow over a stretching sheet with the convective boundary condition at the surface. Vajravelu et al. [17] performed a numerical study of the convective heat transfer of Ag-water and Cu-water nanofluids flow over a non-isothermal stretching sheet. Khan et al. [18] studied the flow and heat transport of ferrofluid over a flat surface subjected to uniform heat flux and slip velocity. Problem of the natural convection of a nanofluid over vertical plate embedded in porous media is investigated by Noghrehabadadi et al. [19]. Rana and Bhargava [20] carried out the numerical study of the flow and heat transfer of a nanofluid over a nonlinearly stretching sheet by using two different methods (finite difference and finite element). Some other related studies have investigated different aspects of nanofluids passing a stretching sheet [21–28].

Recently, nanofluid flows that respond to the imposition of magnetic fields have attracted much attention. Chiam [6] examined magneto-hydrodynamics (MHD) flow over a surface stretching with a power-law velocity using the numerical shooting method. Helmy [7] perused the problem of MHD boundary layer flow for a power law fluid. Very recently, the behavior of boundary layer flow due to the nanofluids movement on a stretching sheet in the presence of magnetic field is evaluated in many articles.

The effects of uniform magnetic field, radiative flux and slip boundary condition are studied on the characteristics of heat transfer and flow of a nanofluid over a permeable stretching sheet by Ibrahim and Shankar [29]. Ferdows et al. [30] carried out an analysis of a nanofluid flow passing a non-linear stretching flat plate in the presence of radiative heat flux and a non-uniform magnetic field. Khan et al. [31] analyzed unsteady boundary layer flow of a nanofluid over a horizontal stretching sheet and reported the effects of thermal radiation and magnetic field on the heat transfer rate (Nusselt number), mass transfer rate (Sherwood number) and shear stress. Mabood et al. [32] have considered MHD boundary layer flow and heat transfer of a nanofluid over a nonlinearly stretching sheet with viscous dissipation effects. Some of the related articles listed in [33–40].

Bioconvection is defined as the macroscopic fluid motion because of the density gradient resulting from collective swimming of motile micro-organisms [41–45]. The self-impelled motile micro-organisms enhance the base fluid density in a particular direction in such a way that they cause the bioconvection flow. Based on cause of impellent, the motile micro-organisms can be classified into different types of micro-organisms including oxytactic or chemotaxis, negative gravitaxis and gyrotactic micro-organisms. The stimulators of these micro-organisms are Oxygen concentration gradient, negative gravity and the displacement between the center of buoyancy and mass, respectively [41]. Unlike the motile micro-organisms, the nanoparticles are not self-impelled and their motion is because of the Brownian motion and thermophoresis effect in nanofluid. The history of studies on the subject of nanofluid bioconvection is not so long. Kuznetsov firstly discussed it in 2010 [46]. At the beginning, he investigated the onset of bioconvection in a horizontal layer filled with a fluid containing both gyrotactic micro-organisms and nanoparticles. After that, he examined the effect of oxytactic micro-organisms on the characteristics of nanofluid flow [47]. Free convection boundary layer regime passing a horizontal flat sheet of a water-based nanofluid containing micro-organisms is studied by Aziz and et al. [48]. Khan and et al. [49] studied the effect of Navier slip and magnetic field on the heat and mass transfer of a nanofluid with presence of gyrotactic micro-organisms over a vertical surface. In another work, Khan et al. [50] conducted a discussion on the natural convection of non-Newtonian nanofluid containing of gyrotactic micro-organisms along a moveless plate in a porous media. Khan and Makinde [51] investigated the MHD boundary layer flow of a water-based nanofluid containing motile gyrotactic micro-organisms along a linearly stretching sheet. The effect of a uniform magnetic field on nanofluid bioconvection passing a permeable vertical sheet is proposed by Mutuku and Makinde [52].

This paper studies the effects of the presence of a non-uniform magnetic field on behavior of water suspension containing nanoparticles and motile gyrotactic micro-organisms passing a nonlinear stretching sheet. In the present study, we have entered a new concept in the problem of boundary layer flow passing a stretching sheet. We have investigated the transport phenomenon in a nanofluid containing self-impelled motile gyrotactic micro-organisms in the presence of non-uniform magnetic field and convective cooling process. The Brownian motion, thermophoresis and convective cooling phenomenon are also analyzed. The aim of the current paper is to expand the studies of Rana and Bhargava [20] and Mabood et al. [32] by considering the simultaneous effects of micro-organisms and non-uniform magneto-hydrodynamics boundary layer flow with viscous dissipation. Numerical solutions are presented and a comparison with the published data (Mabood et al. [32]) is also incorporated in the article to prove the validity. New set of equations are discretized using the finite difference method and have been linearized by employing the Newton’s linearization technique. Then, Numerical results for various physical parameters are expressed graphically and described in detail.

### Problem Definition and Mathematical Modeling

**Fig 1:**
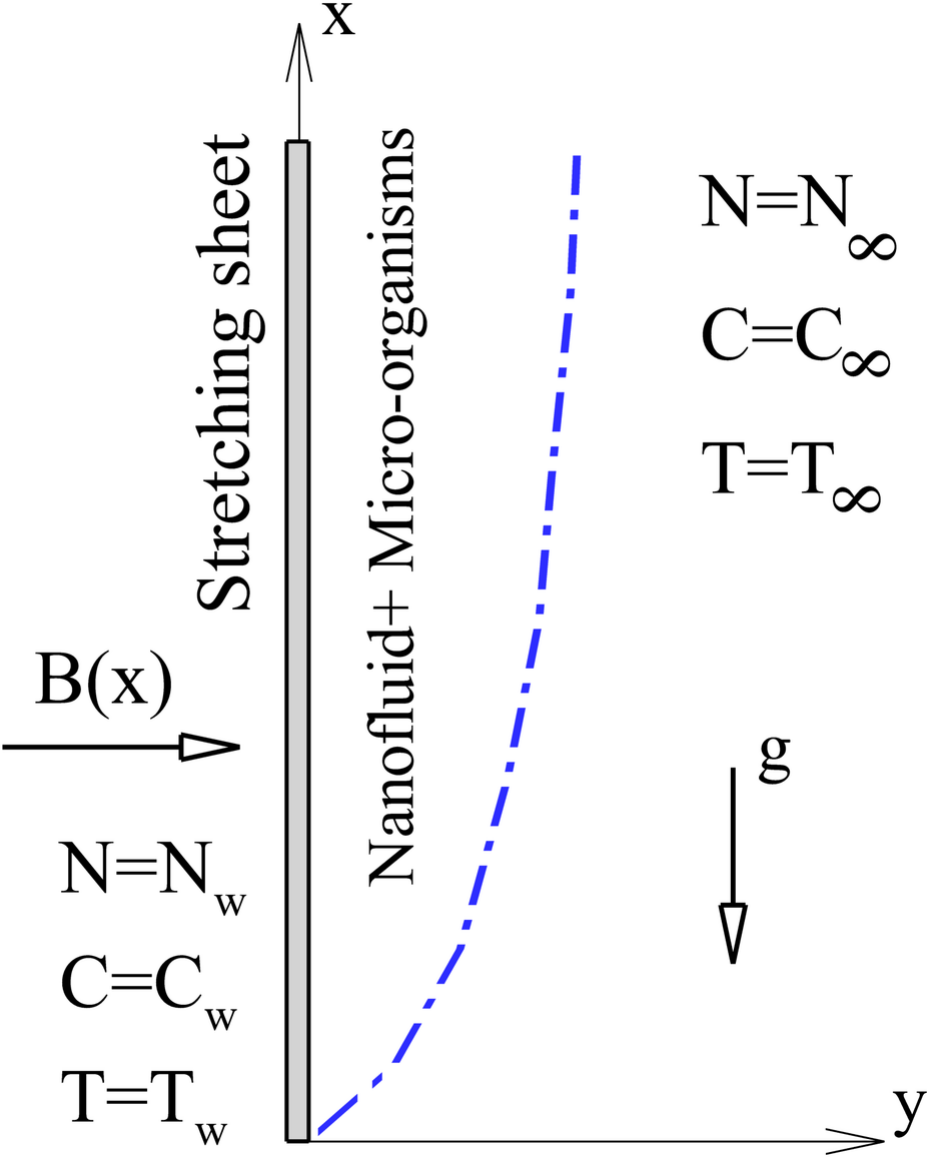
Flow configuration and coordinate system.

We consider a two dimensional, steady, laminar, incompressible viscous boundary layer flow of an electrically conducting nanofluid containing gyrotactic micro-organisms passing a nonlinear stretching vertical flat plate. Water is considered as the base fluid because the micro-organisms only survive in water in natural conditions. Physical model of problem and Cartesian coordinate are shown in Fig 1. During the convection flow, the following assumptions have been considered:

- The sheet is stretching with velocity *u*_*w*_*(x) = ax*^*n*^. *a* is a positive constant and *n* is called the nonlinear stretching parameter.
- The flow field is under influence of a variable magnetic field *B(x)* normal to the stretching sheet and in direction *y* to form *B(x) = B*_*0*_ *x*^*(n-1)/2*^ [6, 30, 32].
- Joule heating is ignored and it is assumed that the induced magnetic field is very small compared to external magnetic field.
- The temperature (*T*_*w*_), nanoparticle concentration (*C*_*w*_) and density of motile microorganism (*N*_*w*_) at the stretching surface are assumed constant and are considered to be greater than the ambient temperature (*T*_*∞*_), nanoparticle concentration (*C*_*∞*_) and density of motile microorganism (*N*_*∞*_), respectively.
- The nanoparticles suspension is stable and dilute such that there is no agglomeration and accumulation of nanoparticles. It should be noted that increasing concentration of nanoparticles leads to the instability.
- It is supposed that the nanoparticles have no effect on the direction and velocity of micro-organism’s swimming.
- Boussinesq approximation is used to determine the variation of density in the buoyancy term.
- Radiative heat transfer is negligible and viscous dissipation is included.
- It is assumed that both the base fluid and nanoparticles locally are in thermal equilibrium state.
- The motile micro-organisms, nanoparticles and base fluid have similar velocity.

Considering the above mentioned assumptions, the governing equations for mass, momentum, energy, nanoparticles concentration and density of gyrotactic micro-organisms can be expressed in the following form [49]:

Continuity,

$$\frac{\partial u}{\partial x}+\frac{\partial v}{\partial y}=0$$ (1)

Momentums,

$$\begin{array}{l} \rho_{f}\left(u\frac{\partial u}{\partial x}+v\frac{\partial u}{\partial y}\right)=-\frac{\partial p}{\partial x}+\mu_{f}\left(\frac{\partial^{2}u}{\partial x^{2}}+\frac{\partial^{2}u}{\partial y^{2}}\right)+\rho_{f}g\beta \left(1-C_{\infty }\right)\left(T-T_{\infty }\right)\\ \;-g\left(\rho_{p}-\rho_{f}\right)\left(C-C_{\infty }\right)-g\gamma \left(\rho_{m}-\rho_{f}\right)\left(N-N_{\infty }\right)-\sigma B_{0}^{2}u \end{array}$$ (2)

$$\frac{\partial p}{\partial y}=0$$ (3)

Energy,

$$u\frac{\partial T}{\partial x}+v\frac{\partial T}{\partial y}=\alpha \left(\frac{\partial^{2}T}{\partial x^{2}}+\frac{\partial^{2}T}{\partial y^{2}}\right)+\tau \left\{D_{B}\frac{\partial C}{\partial y}\frac{\partial T}{\partial y}+\frac{D_{T}}{T_{\infty }}\left({\left(\frac{\partial T}{\partial x}\right)}^{2}+{\left(\frac{\partial T}{\partial y}\right)}^{2}\right)\right\}+\frac{\mu \alpha }{k}{\left(\frac{\partial u}{\partial y}\right)}^{2}+\frac{\sigma \alpha B_{0}^{2}u^{2}}{k}$$ (4)

Nanoparticles concentration,

$$u\frac{\partial C}{\partial x}+v\frac{\partial C}{\partial y}=\alpha \left(\frac{\partial^{2}C}{\partial x^{2}}+\frac{\partial^{2}C}{\partial y^{2}}\right)+\frac{D_{T}}{T_{\infty }}\left(\frac{\partial^{2}T}{\partial x^{2}}+\frac{\partial^{2}T}{\partial y^{2}}\right)$$ (5)

Density of gyrotactic microorganism,

$$u\frac{\partial N}{\partial x}+v\frac{\partial N}{\partial y}+\frac{bW_{c}}{\left(C_{w}-C_{\infty }\right)}\left[\frac{\partial }{\partial y}\left(N\frac{\partial C}{\partial y}\right)+\frac{\partial }{\partial x}\left(N\frac{\partial C}{\partial x}\right)\right]=D_{m}\left(\frac{\partial^{2}N}{\partial x^{2}}+\frac{\partial^{2}N}{\partial y^{2}}+2\frac{\partial^{2}N}{\partial x\partial y}\right)$$ (6)

The pressure terms can be eliminated from the momentum equations by cross-differentiation. Integrating the resulting equation with respect to *y* and using boundary condition at infinity [53], the simplified momentum equation can be written as:

$$\begin{array}{l} \rho_{f}\left(u\frac{\partial u}{\partial x}+v\frac{\partial u}{\partial y}\right)=\mu_{f}\left(\frac{\partial^{2}u}{\partial x^{2}}+\frac{\partial^{2}u}{\partial y^{2}}\right)+\rho_{f}g\beta \left(1-C_{\infty }\right)\left(T-T_{\infty }\right)-g\left(\rho_{p}-\rho_{f}\right)\left(C-C_{\infty }\right)\\ \;-g\gamma \left(\rho_{m}-\rho_{f}\right)\left(N-N_{\infty }\right)-\sigma B_{0}^{2}u \end{array}$$ (7)

The defined boundary conditions for the velocity, temperature, nanoparticles concentration and density of motile micro-organisms fields are as follows:

$$u=ax^{n},\;v=0,\;T=T_{w},\;\varphi =\varphi_{w},\;N=N_{w}\;at\;x=0$$

$$u\to 0,\;v\to 0,\;T\to T_{\infty },\;N\to N_{\infty }\;at\;x\to \infty$$ (8)

In the above equations, *u* and *v* are the velocity components along *x* and *y* axes, respectively, *T* is the temperature, *C* is the nanoparticle concentration, *N* is the density of motile micro-organisms, *p* is the pressure, *ρ*_*f*_, *ρ*_*p*_, *ρ*_*m*_ are the density of nanofluid, nanoparticles and micro-organisms, respectively, *D*_*B*_, *D*_*T*_, *D*_*m*_ are the Brownian diffusion coefficient, thermophoresis diffusion coefficient and diffusivity of micro-organisms, respectively, *k*, *σ* are the thermal and electrical conductivity of the fluid, respectively, *α = k/(ρC*_*p*_*)* is the thermal diffusivity, *γ* is the average volume of a microorganism, *b* is the chemotaxis constant, *W*_*c*_ is maximum cell swimming speed and *bW*_*c*_ is assumed to be constant, *τ = (ρC)*_*p*_*/(ρC)*_*f*_ is the ratio of the effective heat capacitance of the nanoparticle to that of the base fluid.

Introducing the following similarity transformations [20, 32, 52]:

$$\begin{array}{l} \eta ={\left(\frac{a\left(n+1\right)}{2\nu }\right)}^{0.5}yx^{\left(n-1\right)/2},\;u=ax^{n}f^{\prime }\left(\eta \right),\;v=-{\left(\frac{a\nu \left(n+1\right)}{2}\right)}^{0.5}x^{\left(n-1\right)/2}\left(f\left(\eta \right)+\frac{n-1}{n+1}\eta f^{\prime }\left(\eta \right)\right),\\ \theta \left(\eta \right)=\frac{T-T_{\infty }}{T_{w}-T_{\infty }},\;\varphi \left(\eta \right)=\frac{C-C_{\infty }}{C_{w}-C_{\infty }},\;\chi \left(\eta \right)=\frac{N-N_{\infty }}{N_{w}-N_{\infty }} \end{array}$$ (9)

The partial differential equations are converted into the non-linear, coupled and ordinary differential equation as following:

$$f^{\prime \prime \prime }+ff^{\prime \prime }-(\frac{2n}{n+1}){\left(f^{\prime }\right)}^{2}-Mf^{\prime }+(\frac{2}{n+1})(\frac{Gr}{Re^{2}})(\theta -Nr\varphi -Rb\chi )=0$$ (10)

$$\frac{1}{Pr}\theta^{''}+\theta^{\prime }\left(f+Nb\varphi '\right)+Nt{\left(\theta^{\prime }\right)}^{2}+Ec\left[{\left(f^{\prime \prime }\right)}^{2}+M{\left(f^{\prime }\right)}^{2}\right]=0$$ (11)

$$\varphi^{\prime \prime }+Lef\varphi^{\prime }+(\frac{Nt}{Nb})\theta^{\prime \prime }=0$$ (12)

$$\chi^{\prime \prime }+Lbf\chi^{\prime }-Pe[\varphi^{\prime \prime }(\Omega +\chi )+\varphi^{\prime }\chi^{\prime }]=0$$ (13)

Where,

$$\begin{array}{l} Nb=\frac{\tau D_{B}\left(C_{w}-C_{\infty }\right)}{\alpha },\;Nt=\frac{\tau D_{T}\left(C_{w}-C_{\infty }\right)}{\alpha },\;M=\frac{2\sigma B_{0}^{2}}{a\rho \left(n+1\right)},\;\frac{Gr}{Re^{2}}=\frac{\left(g\beta \left(1-C_{\infty }\right)\left(T_{w}-T_{\infty }\right)x^{3}/\nu^{2}\right)}{u_{w}^{2}x^{2}/\nu^{2}},\\ Nr=\frac{\left(\rho_{p}-\rho_{f}\right)\left(C_{w}-C_{\infty }\right)}{\rho \beta \left(1-C_{\infty }\right)\left(T_{w}-T_{\infty }\right)},\;Rb=\frac{\gamma \left(\rho_{m}-\rho_{f}\right)\left(N_{w}-N_{\infty }\right)}{\rho \beta \left(1-C_{\infty }\right)\left(T_{w}-T_{\infty }\right)},\;Pr=\frac{\nu }{\alpha },\;Le=\frac{\nu }{D_{B}},\;Lb=\frac{\nu }{D_{m}},\;\\ Ec=\frac{u_{w}^{2}}{C_{p}\left(T_{w}-T_{\infty }\right)},\;Pe=\frac{bW_{c}}{D_{m}},\;\Omega =\frac{N_{\infty }}{\left(N_{w}-N_{\infty }\right)} \end{array}$$ (14)

The boundary conditions of equations in similarity space can be written as

f(0)=0,f'(0)=1,θ(0)=1,φ(0)=1,χ(0)=1,f'(∞)=0,θ(∞)=0,φ(∞)=0,χ(∞)=0 (15)

The prims indicate the derivative with respect similarity variable *η*. In Eqs (10–14), *M* refers to magnetic number, *Gr/Re*^*2*^ is local Richardson number, *Nr* is the buoyancy ratio parameter, *Rb* is the bioconvection Rayleigh number, *Pr* is Prandtl number, *Nb* is the Brownian motion parameter and *Nt* is the thermophoresis parameter, *Ec* is local Eckert number, *Le* and *Lb* are the traditional Lewis number and the bioconvection Lewis number, respectively, *Pe* is the bioconvection Peclet number and *Ω* is the micro-organisms concentration difference parameter. It is necessary to mention that Eqs (10) and (11) are locally similar because the dimensional parameters of Richardson number and Eckert number are local similarity parameters. On the other hand, existence of *Ec* in energy equation makes this equation be the local similarity for all values of *n*, while the momentum equation reduces to a full similarity equation for *n* = 1/2.

The shear stress, the local heat flux, the local mass flux and the motile micro-organisms flux on the surface are *τ*_*w*_, *q*_*w*_, *q*_*m*_ and *q*_*n*_, respectively and can be expressed as

$$\tau_{w}=\mu {\left(\frac{\partial u}{\partial y}\right)}_{y=0},\;q_{w}=-k{\left(\frac{\partial T}{\partial y}\right)}_{y=0},\;q_{m}=-D_{B}{\left(\frac{\partial C}{\partial y}\right)}_{y=0},\;q_{n}=-D_{m}{\left(\frac{\partial N}{\partial y}\right)}_{y=0}$$ (16)

In the present study, the important parameters of the skin friction coefficient *C*_*fx*_, the local Nusselt number *Nu*_*x*_, the local Sherwood number *Sh*_*x*_ and the local density number of the motile micro-organisms *Nn*_*x*_ are defined as

$$C_{fx}=\frac{\tau_{w}}{\rho u_{w}^{2}},\;Nu_{x}=\frac{xq_{w}}{k\left(T_{w}-T_{\infty }\right)},\;Sh_{x}=\frac{xq_{m}}{D_{B}\left(C_{w}-C_{\infty }\right)},\;Nn_{x}=\frac{xq_{n}}{D_{m}\left(N-N_{\infty }\right)}$$ (17)

By combining the Eq (14) and Eq (15), we obtain

$$Re_{x}^{1/2}C_{fx}=\sqrt{\frac{n+1}{2}}f^{\prime \prime }\left(0\right),\;Re_{x}^{-1/2}Nu_{x}=-\sqrt{\frac{n+1}{2}}\theta^{\prime }\left(0\right),\;Re_{x}^{-\frac{1}{2}}Sh_{x}=-\sqrt{\frac{n+1}{2}}\varphi^{\prime }\left(0\right),\;Re_{x}^{-1/2}Nn_{x}=-\sqrt{\frac{n+1}{2}}\chi^{\prime }\left(0\right)$$ (18)

In the above equation, *Re*_*x*_ *= U*_*0*_*x/ν* refers to the local Reynolds number.

### Numerical Solution Technique

The system of non-linear, coupled and ordinary differential Eqs (10–13) subjected to the boundary conditions (15) has been solved using the iterative finite difference method. Before discretizing the ordinary Eqs (10–13), first, Eq (10) is simplified to a set of equation as follows;

$$\left\{ \begin{array}{l} f^{\prime }=z\\ z^{\prime \prime }+fz^{\prime }-z^{2}+Gr\left(\theta -Nr\varphi -Rb\chi \right)=0 \end{array}\right.$$ (19-a), (19-b)

**Fig 2:**
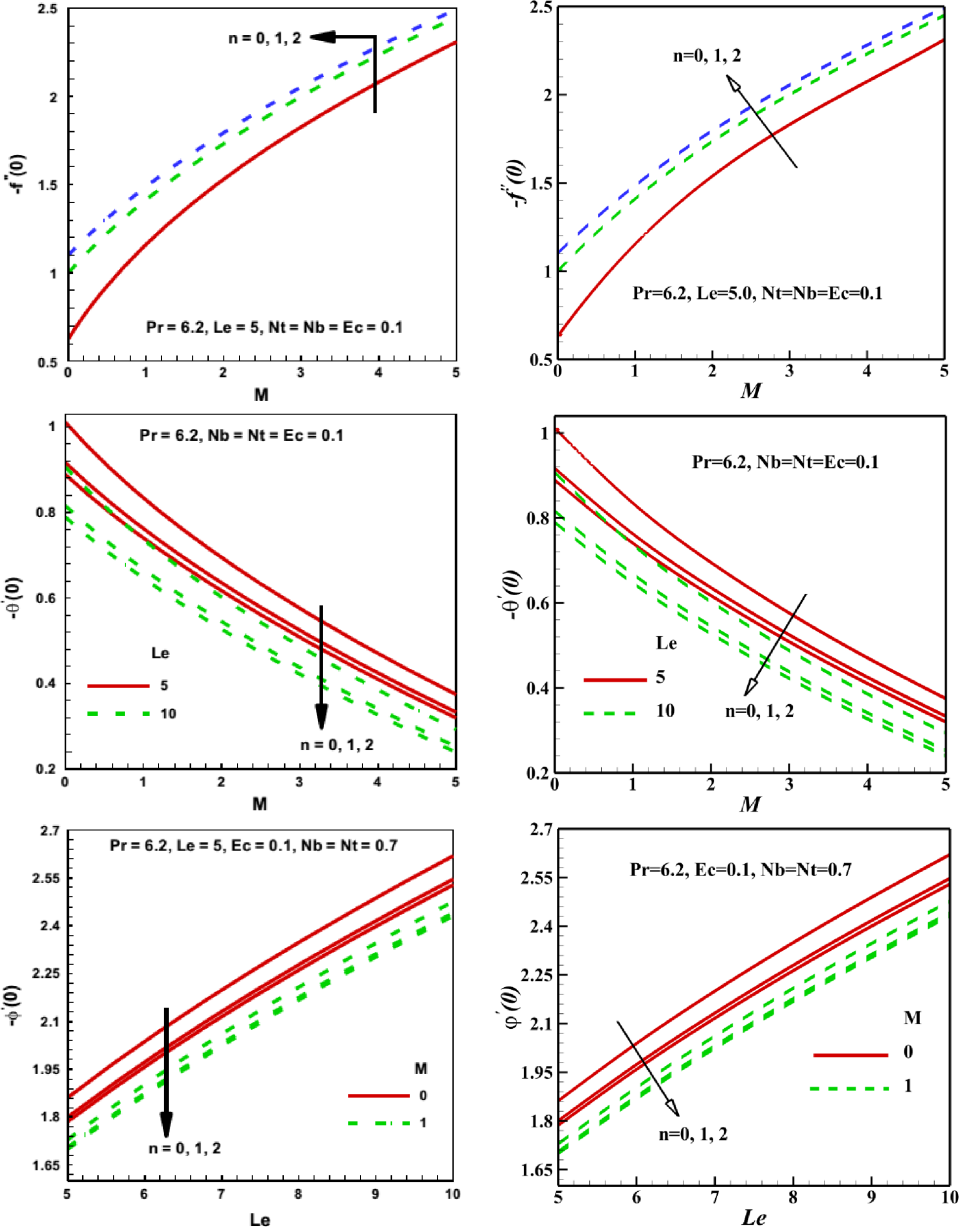
Effects of different parameters on the skin friction coefficient (*-f*^*"*^*(0)*), Nusselt number (*-*θ'(0)) and Sherwood number (*-*φ'(0)) (a) Present code and (b). Mabood et al. [32].

As the equations show, the momentum equation is coupled with heat and nanoparticles concentration equations by the buoyancy term in the momentum equation. So, it is necessary to solve these equations in the coupled form. First, third order momentum equation is simplified into two (one and two order) equations, because the coefficient matrix must be three dimensional at the numerical method used to solve the algebraic equations. Then, these two equations are simultaneously solved with the other governing equations. Eq (19-a) is discretized using backward difference approximation because the exact value of *f* is given in the first node. Eqs (11–13) and (19-b) are discretized using the central difference approximation and the nonlinear terms linearized by Newton’s method. Thus, the differential Eqs (11–13) and (19-b) are converted into a tri-diagonal system of algebraic equations that can be solved by the well-known Thomas algorithm. The step size and error tolerance have been considered 10^−4^ and 10^−6^, respectively. Results show that the choice of η_∞_ = 12 satisfies the perfect effect of boundary layers. To ensure the accuracy and validity of the present solution, we have compared the obtained results from our program with the ones published in the literature for the skin friction coefficient, Nusselt and Sherwood numbers in Table 1 (Khan and Pop [24]), Fig 2 (Mabood et al. [32]). In another validation, results obtained by this study and those reported by Akbar and Khan [54] are compared. Results of this comparison are reflected in Table 2. As it is noticed, there is excellent agreement between the results of the present study and those published in the literature [24, 32, 54], so we are confident to use the present code.

**Table 1:** Comparison of Nusselt and Sherwood numbers between the results of present study and reported by Khan and Pop [24] at *Le* = *Pr* = 10, *n* = 1, *M* = *Ec* = *Nr* = *Rb* = *Pe* = *Lb* = *Ω* = 0 and different values for *Nt* and *Nb*.

*Nb*	*Nt*	-θ'(0)		-φ'(0)	
		Present code	Khan and Pop [24]	Present code	Khan and Pop [24]
0.1	0.1	0.952493	0.9524	2.129151	2.1294
	0.2	0.693282	0.6932	2.273639	2.2740
	0.3	0.520173	0.5201	2.528152	2.5286
	0.4	0.402662	0.4026	2.794607	2.7952
	0.5	0.321125	0.3211	3.034519	3.0351
0.3	0.1	0.252242	0.2522	2.409896	2.4100
	0.2	0.181664	0.1816	2.514845	2.5150
	0.3	0.135567	0.1355	2.608648	2.6088
	0.4	0.104652	0.1046	2.687421	2.6876
	0.5	0.083334	0.0833	2.751676	2.7519
0.5	0.1	0.054284	0.0543	2.383477	2.3836
	0.2	0.039063	0.0390	2.446704	2.4468
	0.3	0.029153	0.0291	2.498260	2.4984
	0.4	0.022513	0.0225	2.539748	2.5399
	0.5	0.017933	0.0179	2.572979	2.5731

**Table 2:** Comparison of the density number of the motile micro-organisms between the results of present study and reported by Akbar and Khan [54] at *n* = 1, *M* = Nb = 1, *Ec* = 0, *Nr* = Nt = Gr = 0.5, Lb = Le = 2, *Rb* = 0.3 and different values for *Ω* and *Pe*.

*Ω*	*Pe* = 0.3		*Pe* = 0.5		*Pe* = 0.7	
	Akbar and Khan [54]	Present code	Akbar and Khan [54]	Present code	Akbar and Khan [54]	Present code
0.1	2.772	2.819	3.143	3.203	3.525	3.598
0.2	2.814	2.861	3.215	3.275	3.624	3.699
0.4	2.894	2.945	3.353	3.418	3.823	3.903
0.6	2.978	3.029	3.494	3.561	4.025	4.107
0.8	3.059	3.114	3.636	3.704	4.232	4.311
1.0	3.143	3.197	3.773	3.847	4.434	4.515

## Results and Discussion

### Velocity Profiles

**Fig 3:**
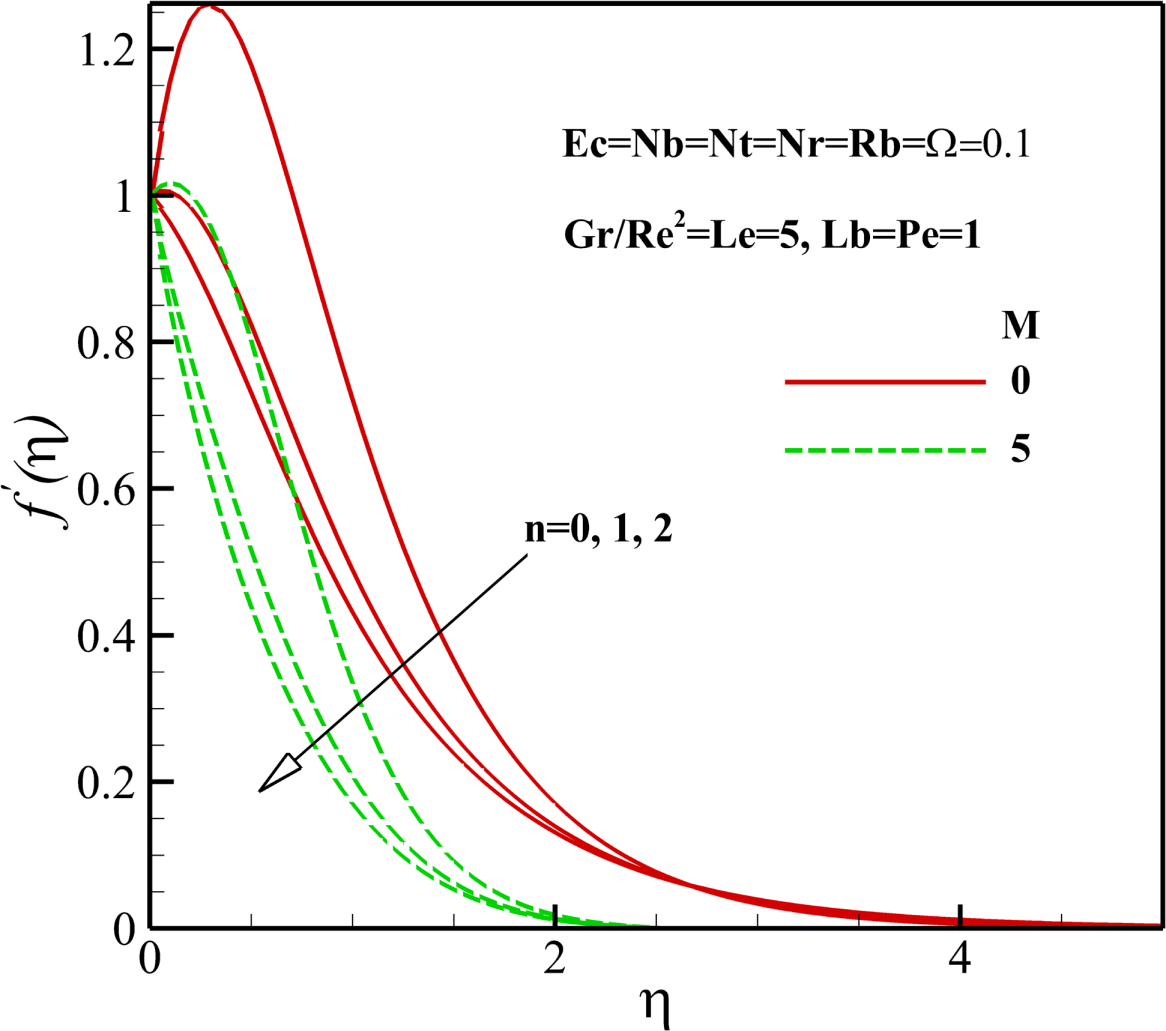
Effects of *M* and *n* on the dimensionless velocity.

**Fig 4:**
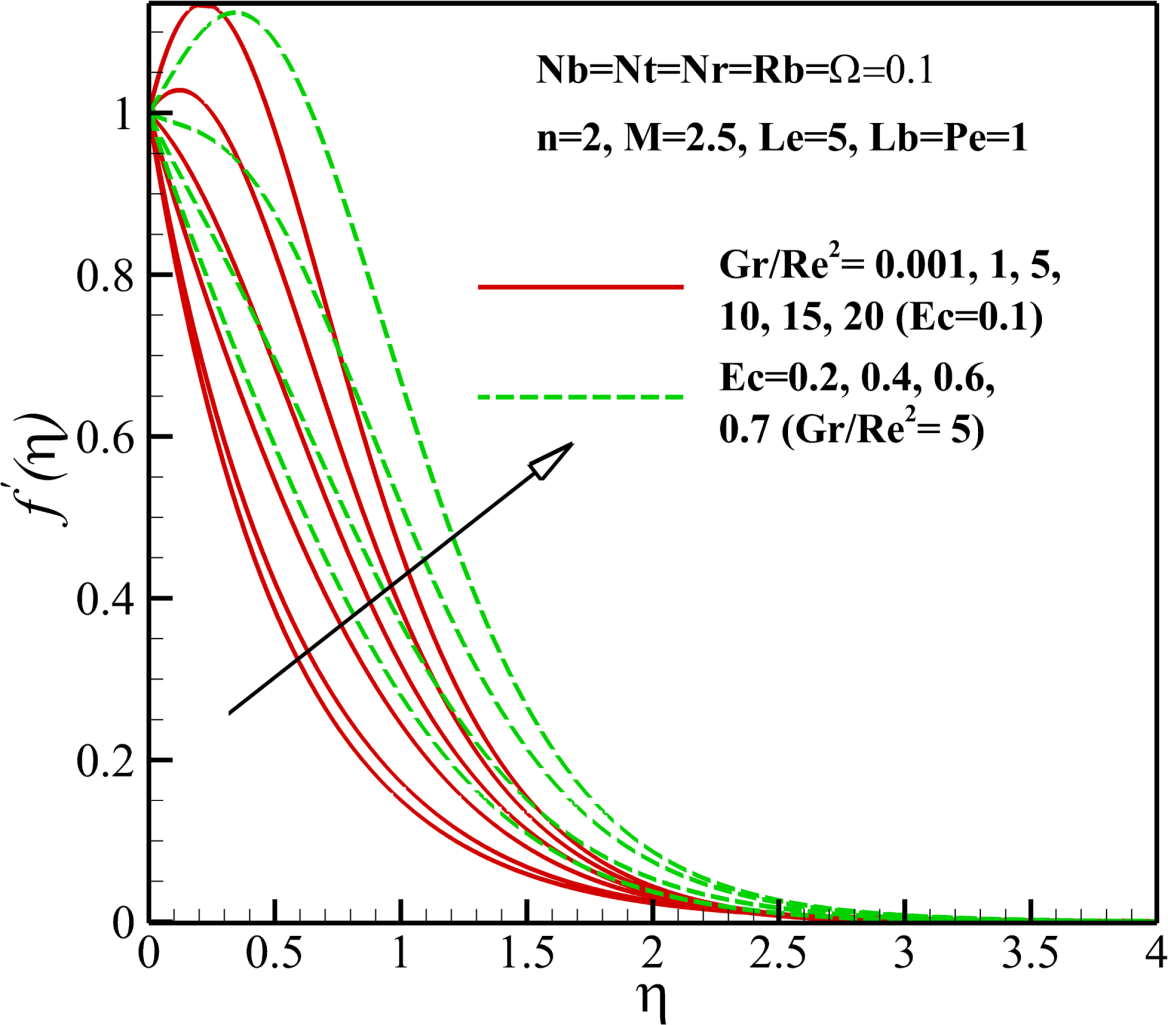
Effects of *Gr/Re*^*2*^ and *Ec* on the dimensionless velocity.

**Fig 5:**
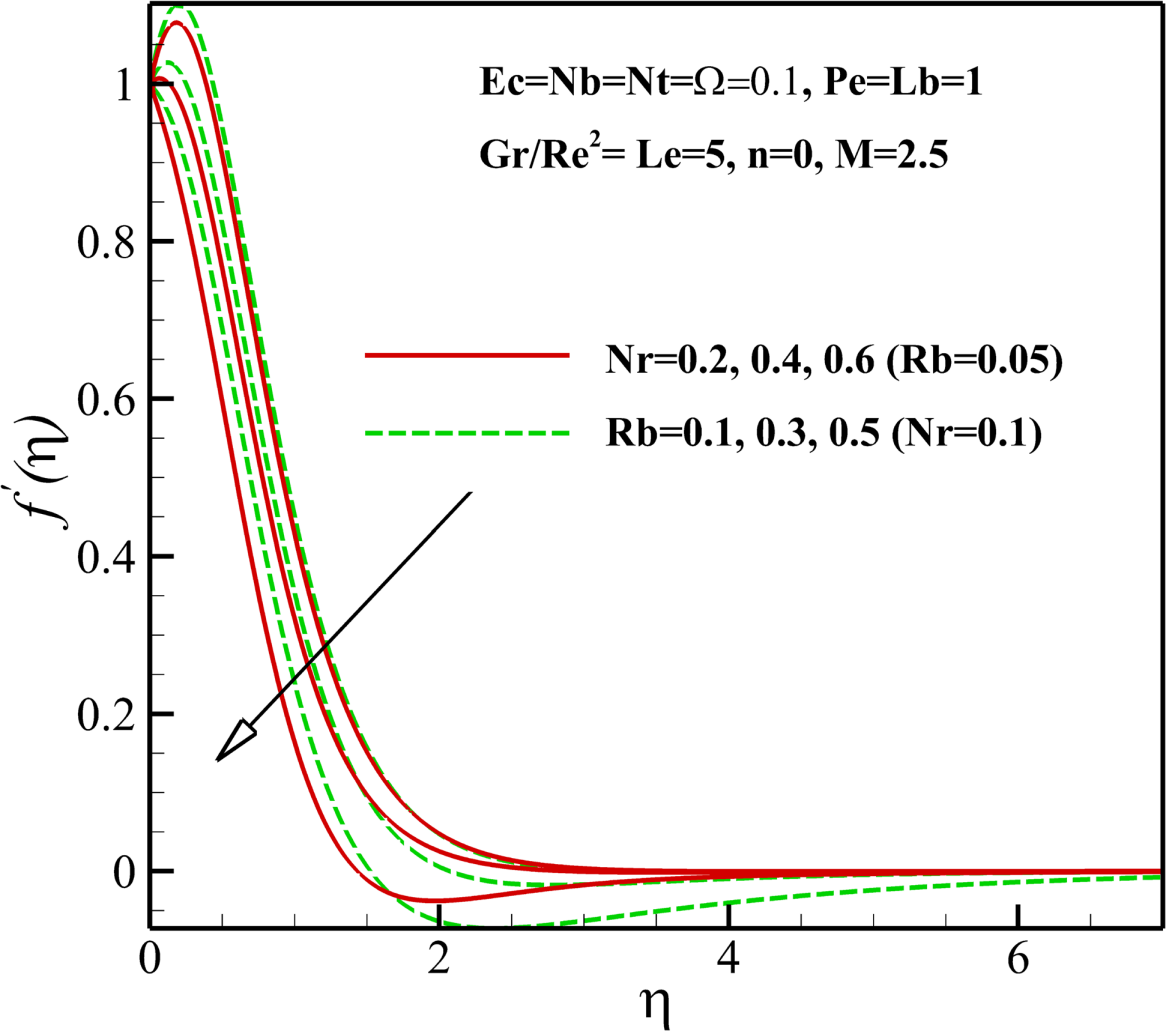
Effects of *Nr* and *Rb* on the dimensionless velocity.

Figs 3–5 show the effects of various parameters on the dimensionless velocity of nanofluid flow. The set of these figures tells us that based on the values of dimensionless variables, the maximum dimensionless velocity occurs at the sheet surface or at various distances from it. The velocity overshoot in the adjacency of the sheet is the result of the buoyancy force. The presence of body force induced by magnetic field, known as Lorentz force, leads to the deceleration of momentum and accordingly, causes a decrease in the velocity overshoot and momentum boundary layer thickness as represented in Fig 3. As shown in Fig 4, the velocity increases with enhancing Eckert number *Ec*. This result can be justified by this explanation that heating due to viscous dissipation of the fluid increases the resulting increases in *Ec*. On the other hand, we know that heating due to viscous dissipation reduces the viscosity of the nanofluid which results in increasing locomotion. According to Eq (8), it can be said that the effect of buoyancy force increases and decreases with an increasing in Richardson number *Gr/Re*^*2*^ and nonlinear stretching parameter n, respectively, thus it is clear that the velocity increases or overshoots in the adjacency of the sheet as *Gr/Re*^*2*^ increases and n decreases as shown in Figs 3 and 4. The implication of increasing *Rb*, is that the power of convection caused by bioconvection is enhanced against the convection of buoyancy force. Thus, it can be stated that the flow velocity decreases with increasing in *Rb* as displayed in Fig 5. Also, from Fig 5, it can be seen that the dimensionless velocity decreases with increasing *Nr* due to increase in the negative buoyancy created by the presence of nanoparticles.

### Temperature Profiles

**Fig 6:**
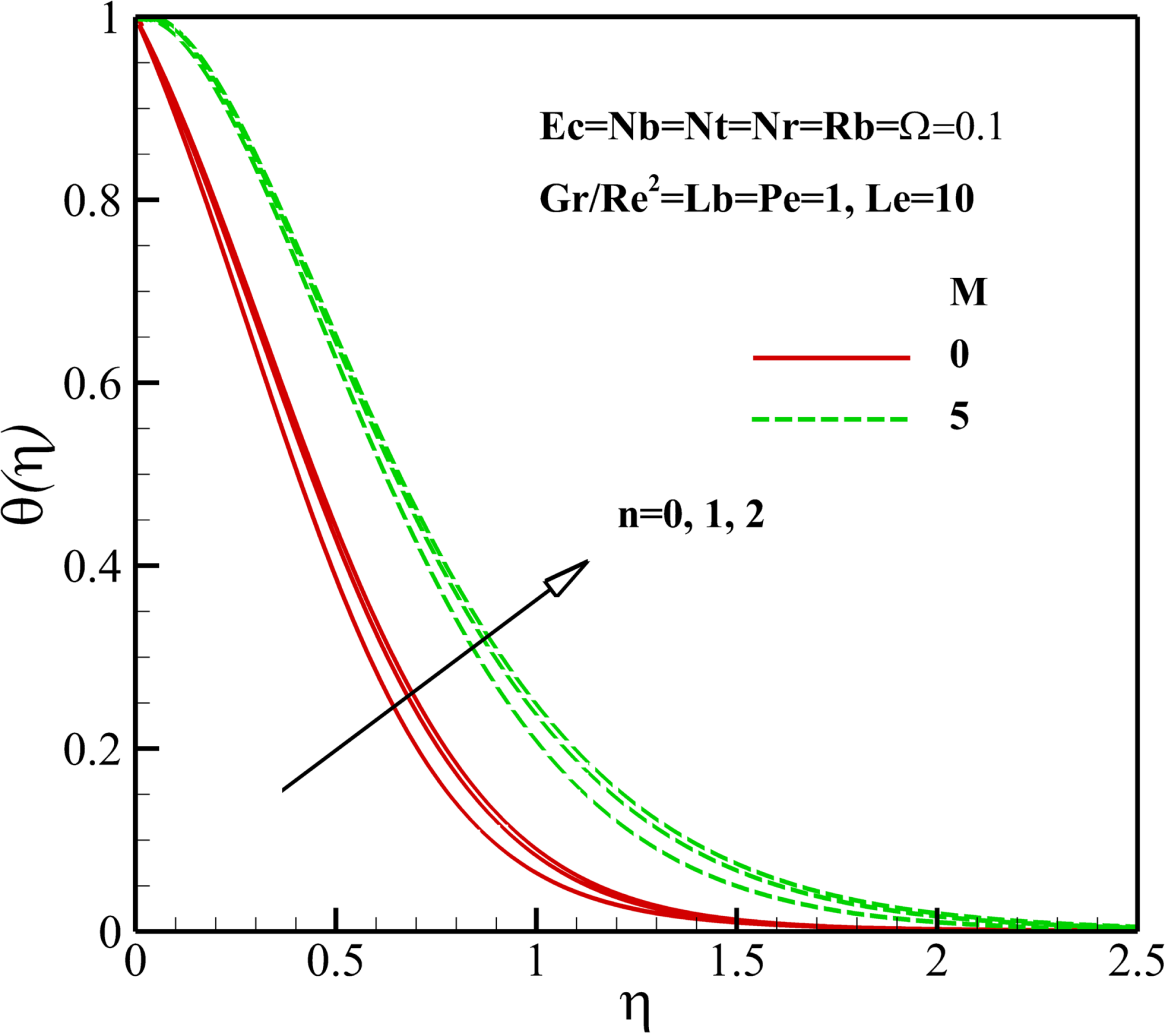
Effects of *M* and *n* on the dimensionless temperature.

**Fig 7:**
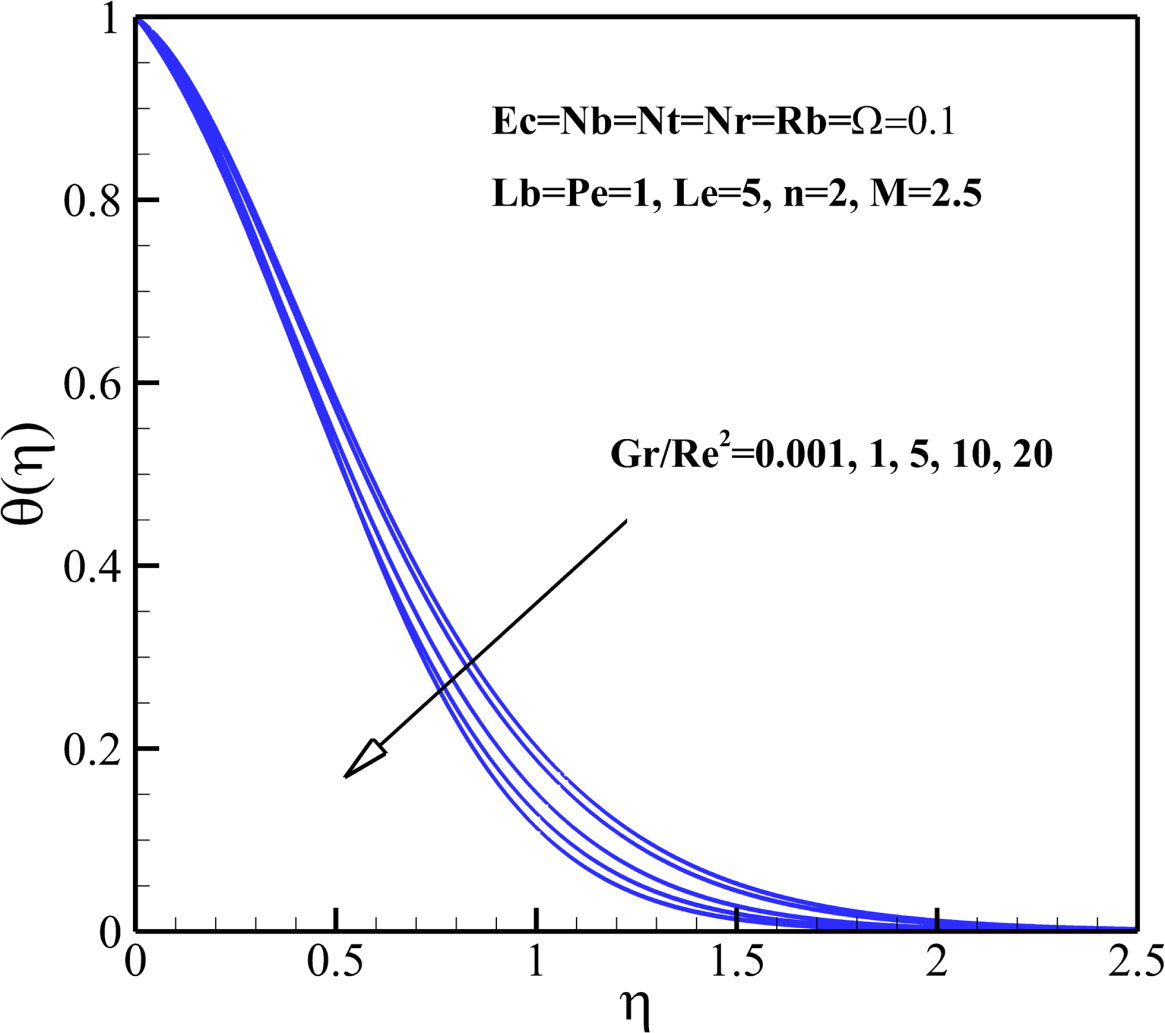
Effects of *Gr/Re*^*2*^ on the dimensionless temperature.

**Fig 8:**
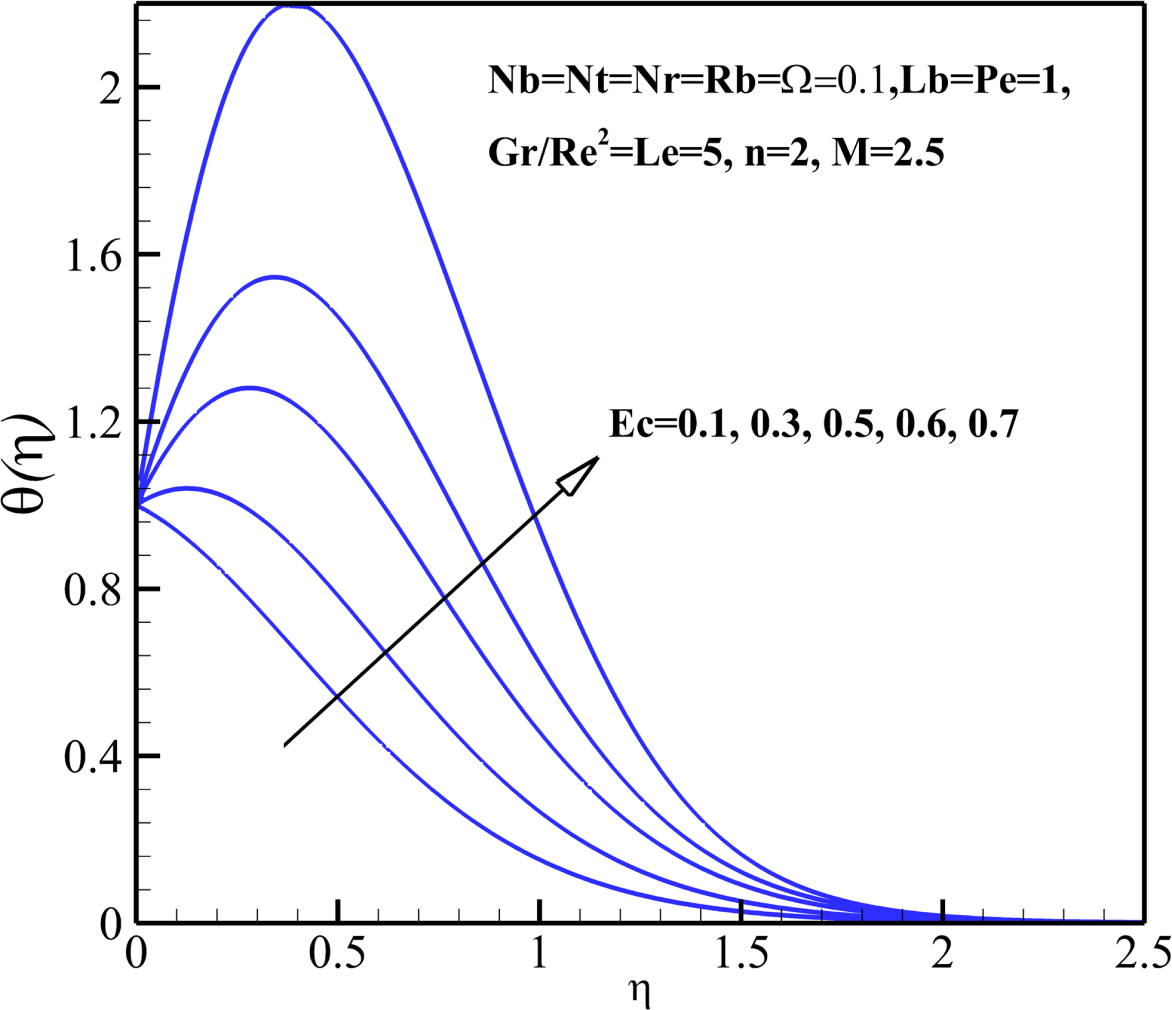
Effects of *Ec* and *Gr/Re*^*2*^ on the dimensionless temperature.

**Fig 9:**
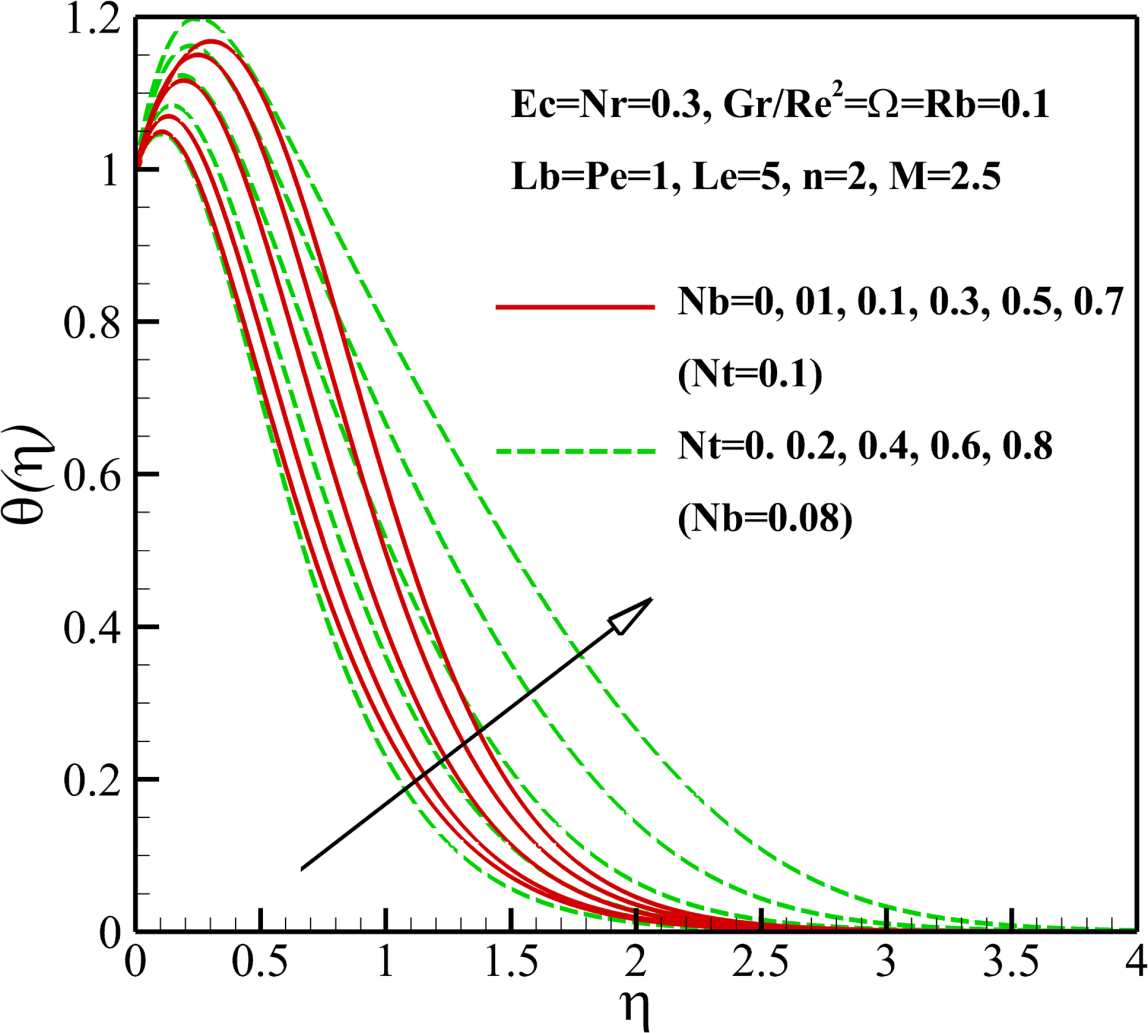
Effects of *Nb* and *Nt* on the dimensionless temperature.

**Fig 10:**
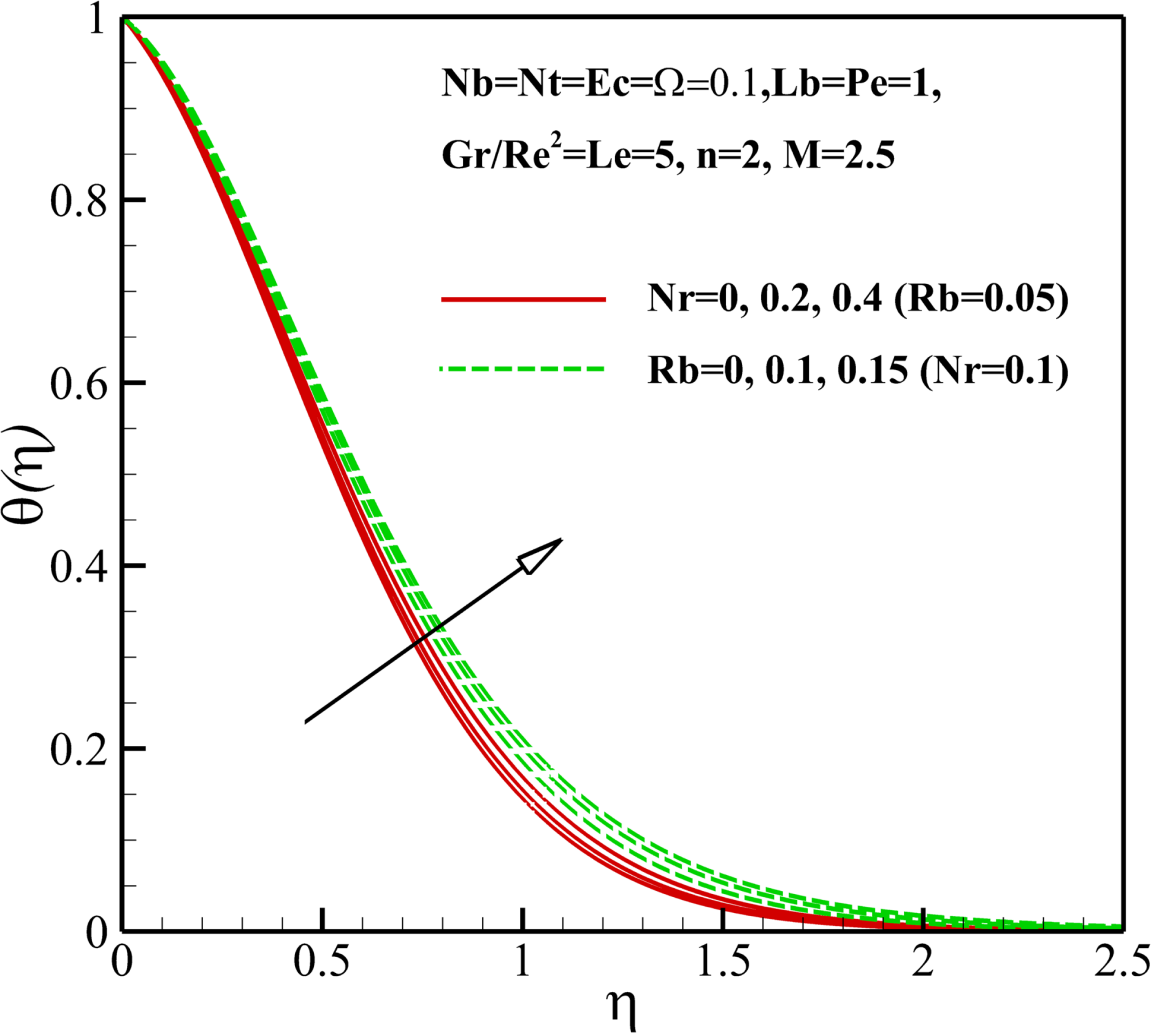
Effects of *Nr* and *Rb* on the dimensionless temperature.

The variation of the dimensionless temperature within the thermal boundary layer with thermophysical parameters are demonstrated in Figs 6–10. The dimensionless temperature increases due to a decrease in the dimensionless velocity with increasing magnetic field *M* and the nonlinear stretching parameter *n* As shown in Fig 6, it can be seen that the effect of magnetic parameter *M* on the thermal boundary layer thickness is much more pronounced than the nonlinear stretching parameter *n*. Compared to the magnetic field, there are the revers conditions for Richardson number *Gr/Re*^*2*^ as illustrated in Fig 7. As shown in Figs 8 and 9, both the dimensionless temperature and thermal boundary layer thickness increase with the increase of thermophoresis parameter *Nt*, Brownian motion parameter *Nb* and Eckert number *Ec*. The additional heating that is created by the interaction of nanoparticles and the fluid due to the Brownian motion, thermophoresis effect and viscous dissipation increases the temperature. Consequently, the thermal boundary layer thickness becomes thicker for the values of higher *Nb*, *Nt* and *Ec*. Also, it can be expressed that due to the additional heating the dimensionless temperature overshoots in the vicinity of the stretching sheet. Fig 10 demonstrates that bioconvection Rayleigh number *Rb* and buoyancy ratio parameter *Nr* slightly enhance the dimensionless temperature of nanofluid.

### Nanoparticles Concentration Profiles

**Fig 11:**
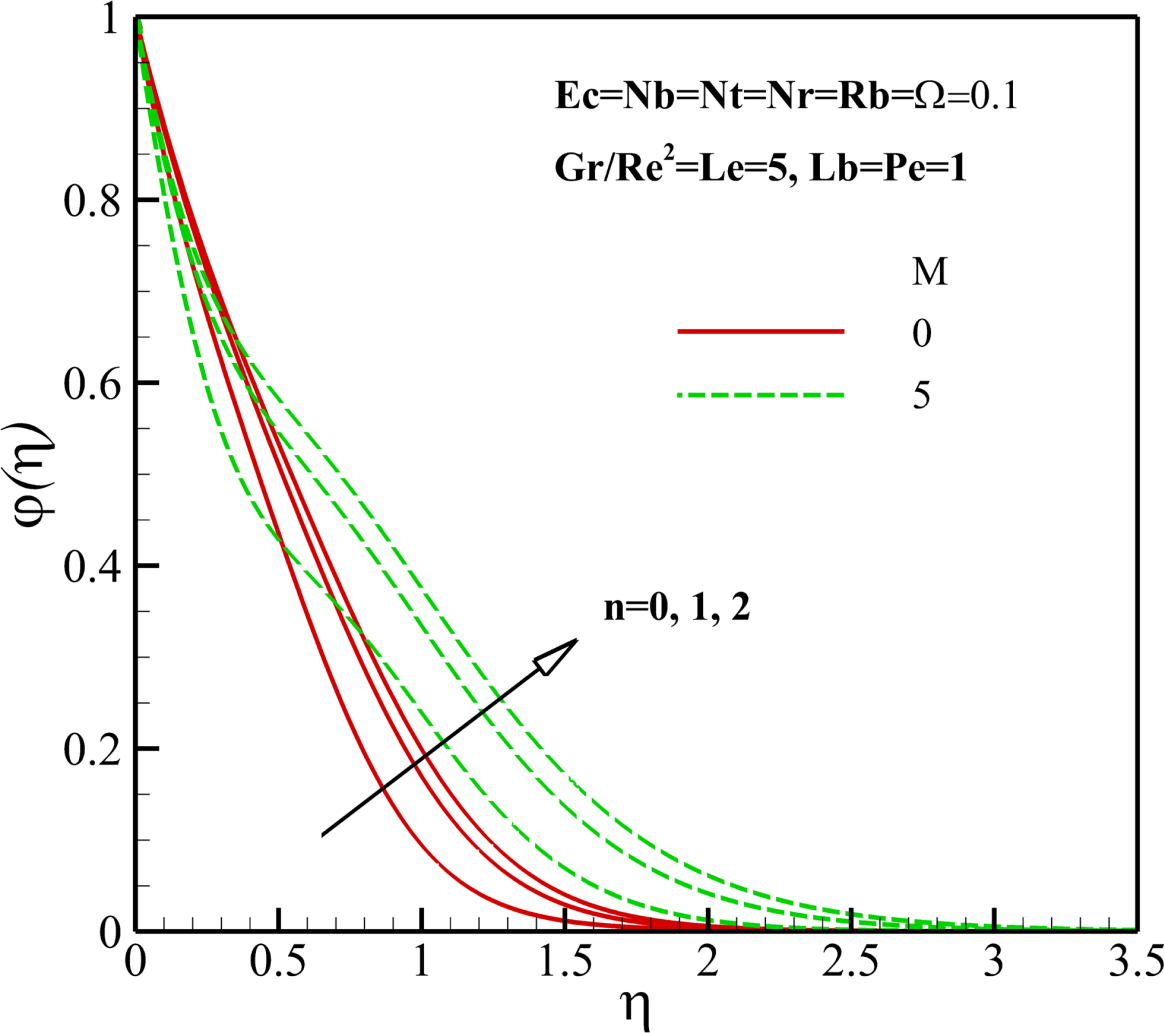
Effects of *M* and *n* on the nanoparticles concentration.

**Fig 12:**
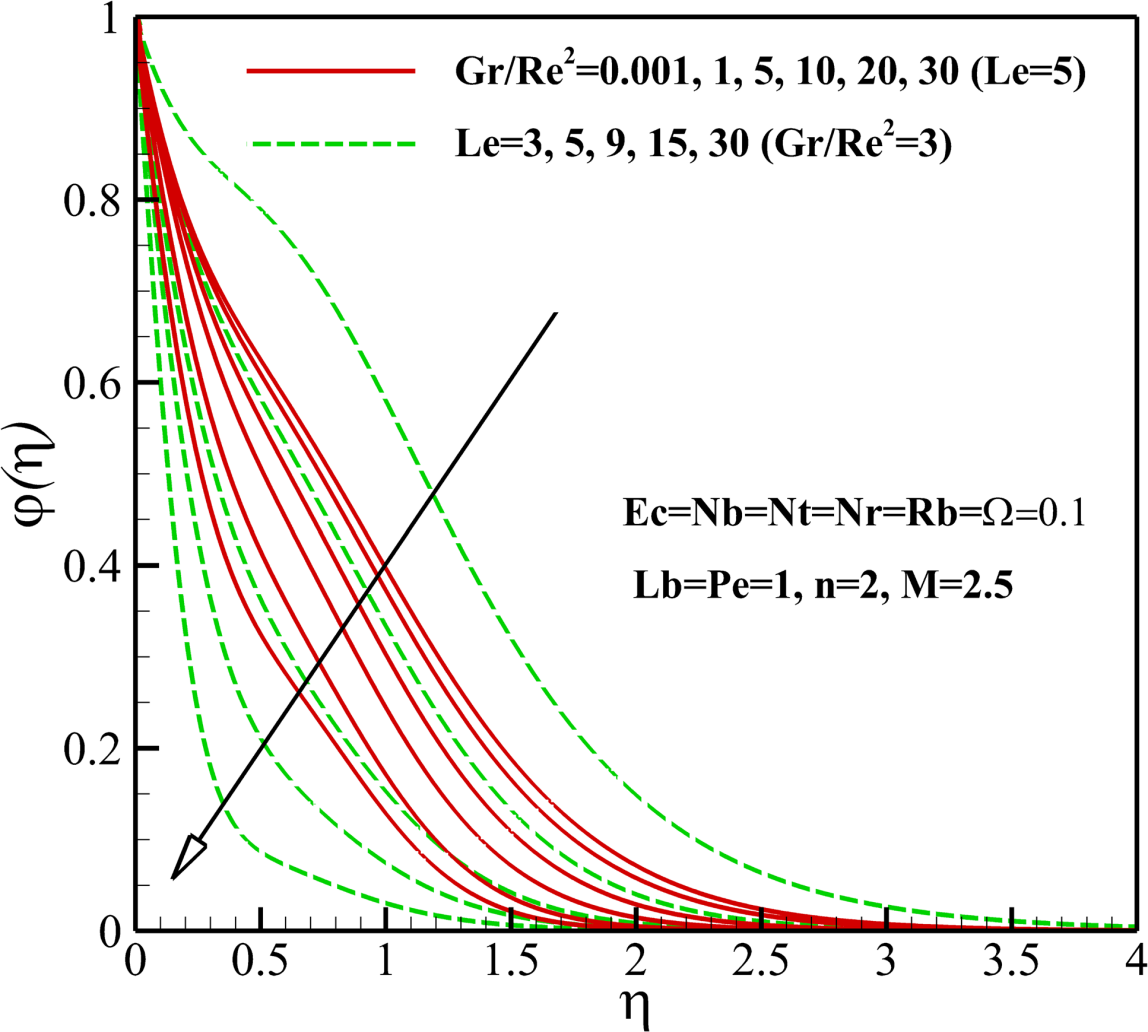
Effects of *Gr/Re*^*2*^ and *Le* on the nanoparticles concentration.

**Fig 13:**
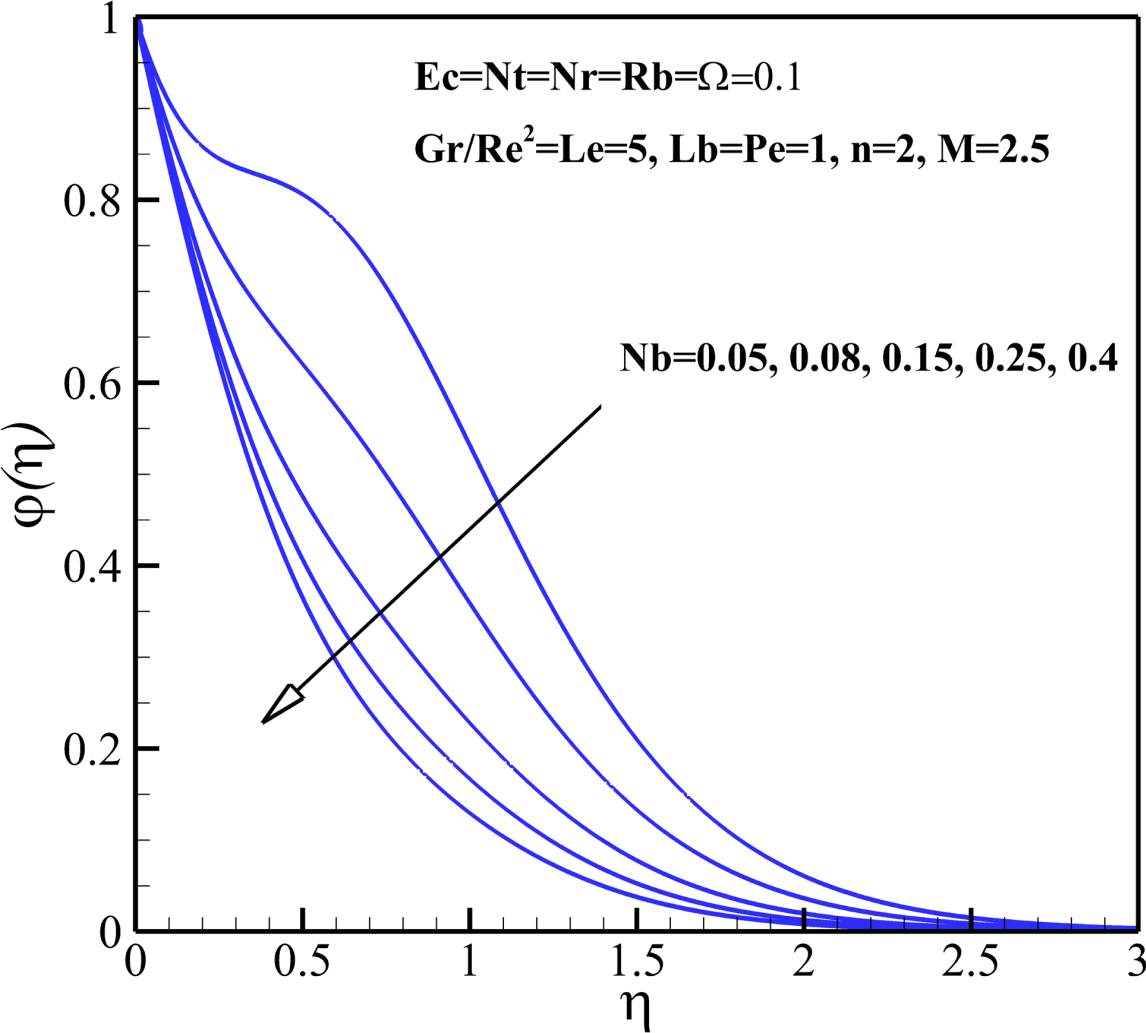
Effects of *Nb* on the nanoparticles concentration.

**Fig 14:**
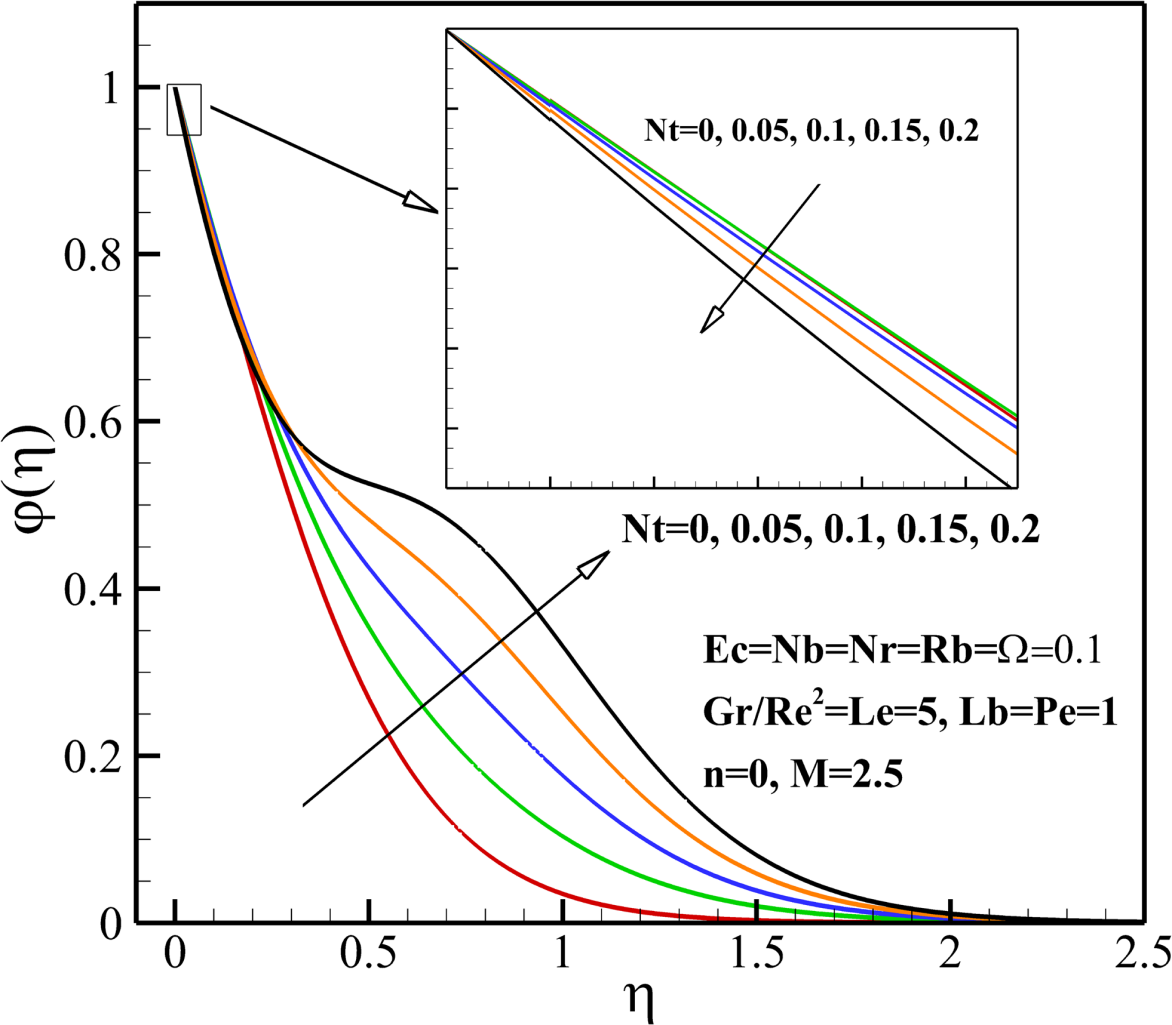
Effects of *Nt* on the nanoparticles concentration.

Figs 11–14 display the influence of the different parameters on the nanoparticles concentration. As depicted in Figs 11 and 12, the nanoparticles concentration, within the concentration boundary layer, increases and decreases with an increase of nonlinear stretching parameter n and Richardson number *Gr/Re*^*2*^, respectively. This is due to the fact that the dimensionless velocity decreases and increases as growing of *n* and *Gr/Re*^*2*^, respectively. Also, it is important to note that the nanoparticles concentration decreases near the flat plate and increases away from it with an increase in magnetic parameter *M*. Thus, it is clear that the momentum boundary layer thickness becomes thicker with *n* and *M* and becomes thinner with *Gr/Re*^*2*^.

Both the nanoparticles concentration and boundary layer thickness significantly decreases with increasing Lewis number *Le* as depicted in Fig 12. This is due to the fact that the convection of nanoparticles increases as Lewis number *Le* increases. From Fig 13, it is can be seen that both the nanoparticles concentration and boundary layer thickness decreases with increasing Brownian motion parameter *Nb*. Fig 14 shows that The nanoparticles concentration increases in the vicinity of stretching sheet and decreases far from it as thermophoresis parameter *Nt* increases. So, it can be concluded that the nanoparticles boundary layer thickness becomes thicker with *Nt*.

### Density of Motile Micro-Organisms Profiles

**Fig 15:**
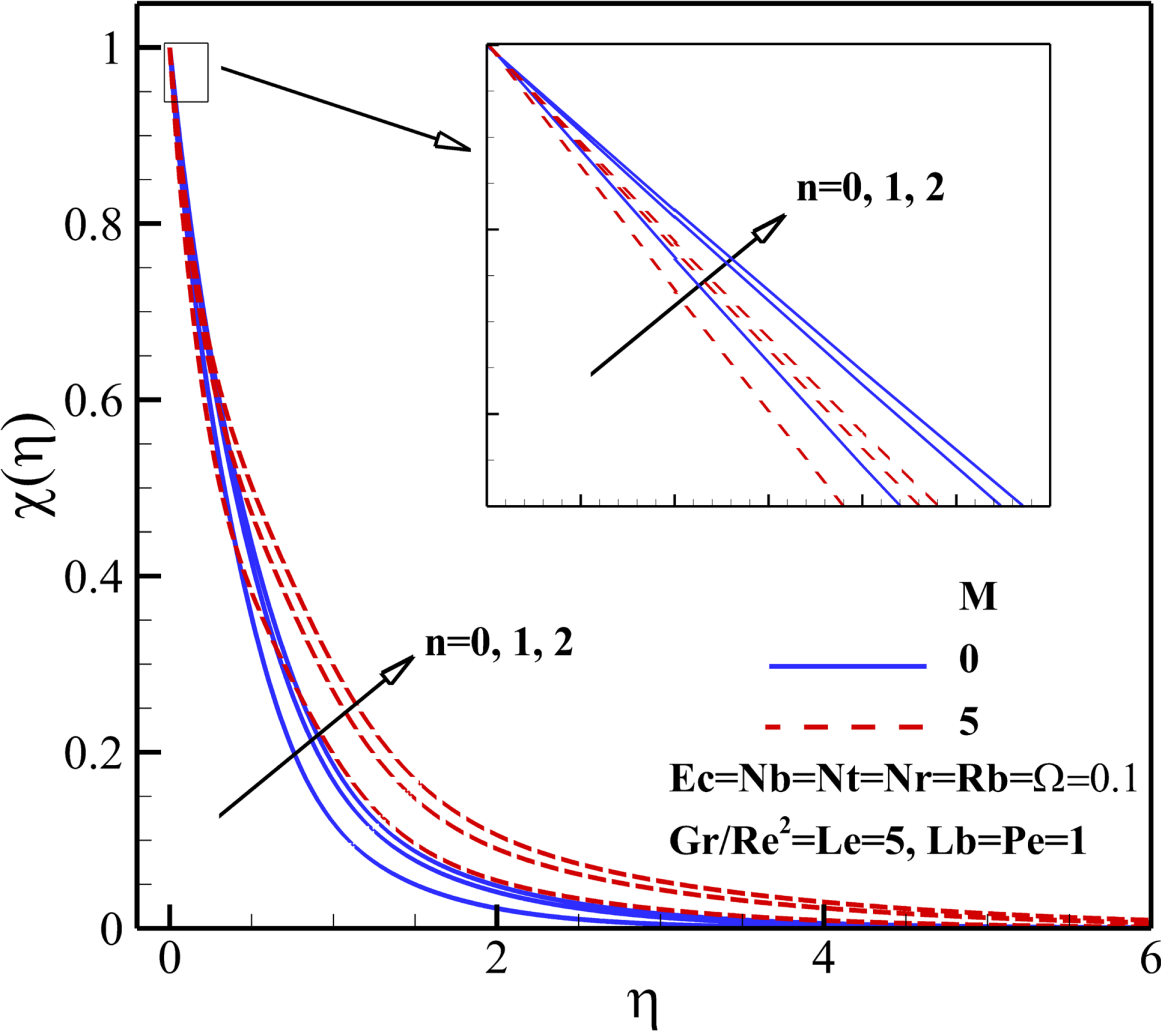
Effects of *M* and *n* on the density of motile microorganisms.

**Fig 16:**
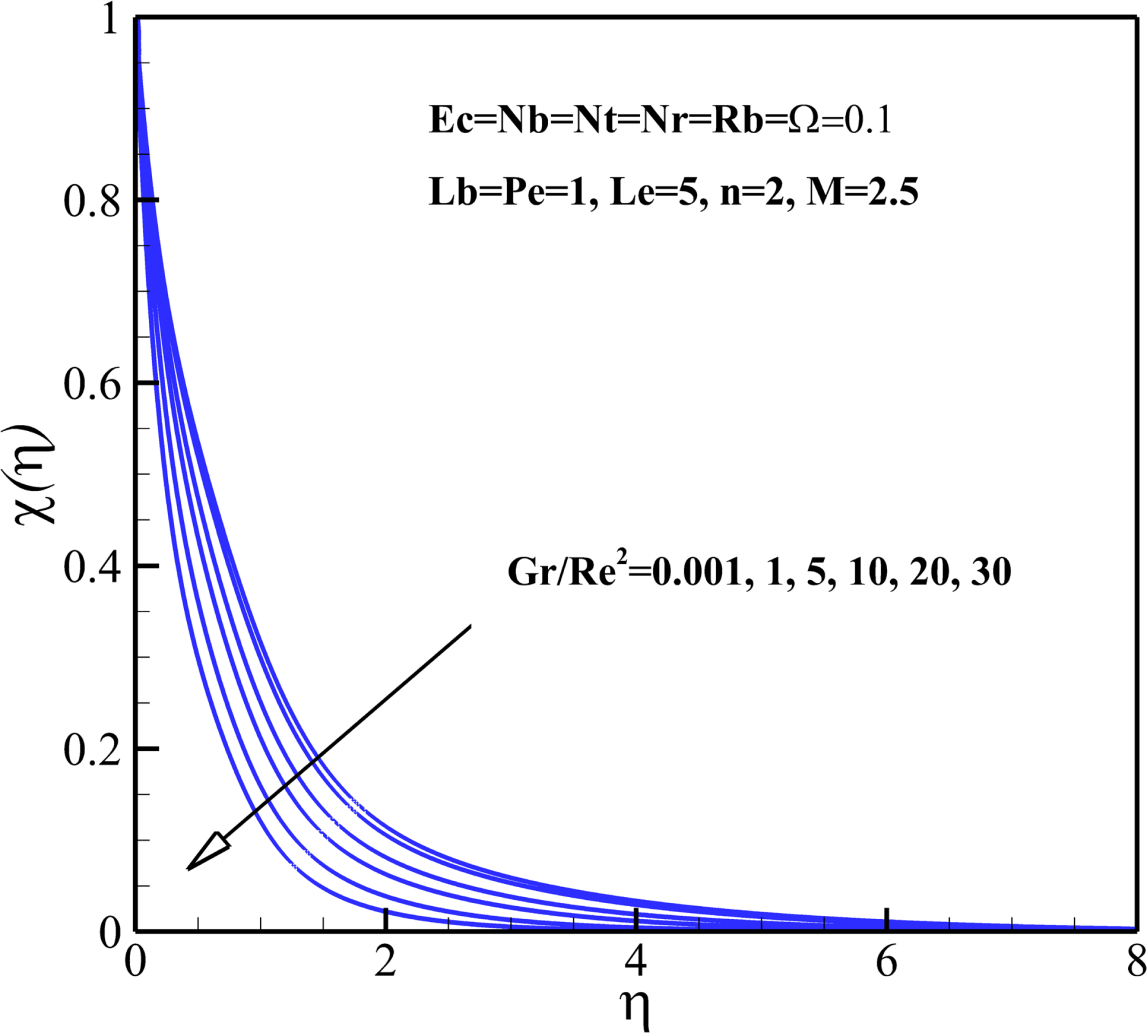
Effects of *Gr/Re*^*2*^ on the density of motile microorganisms.

**Fig 17:**
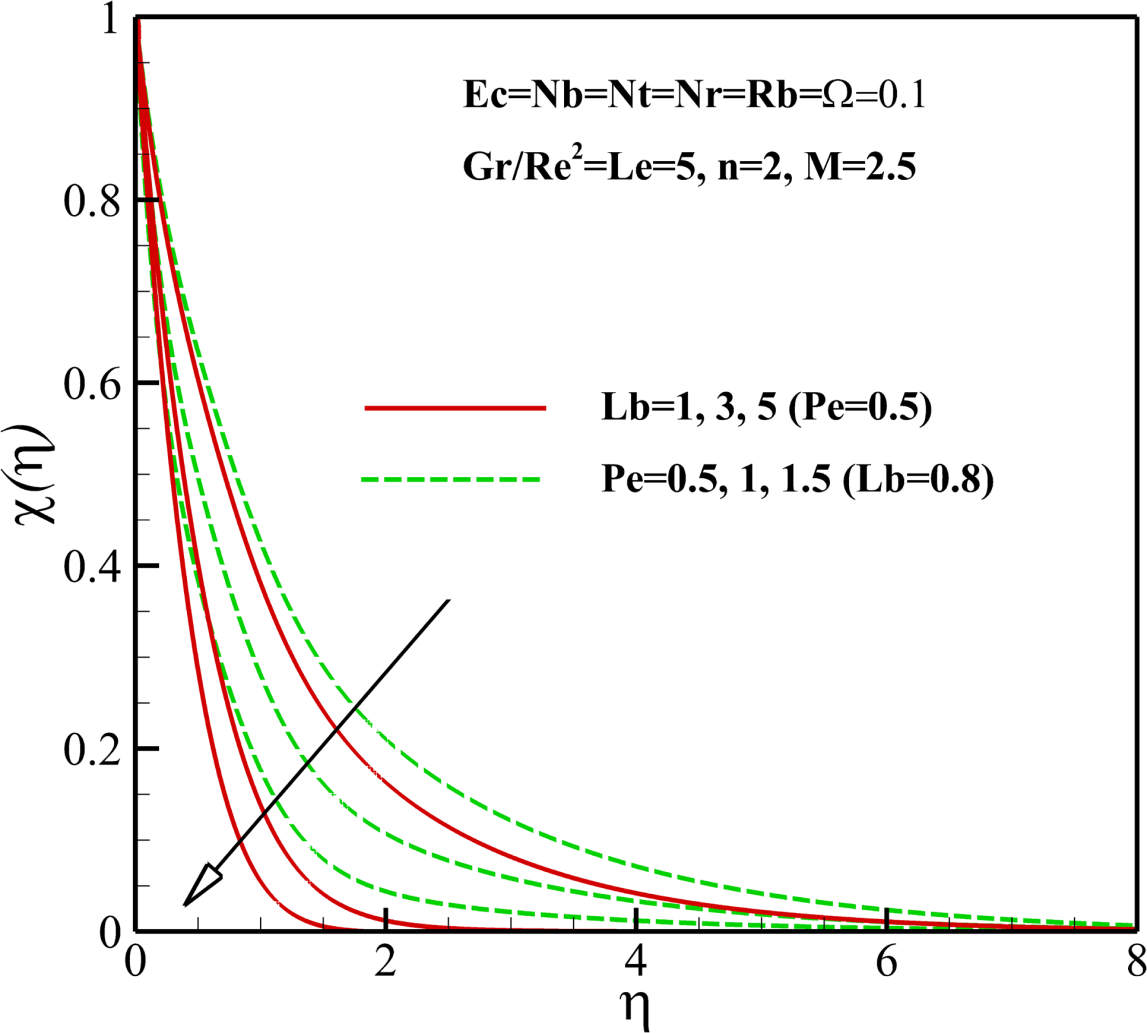
Effects of *Ω* and *Lb* on the density of motile microorganisms.

**Fig 18:**
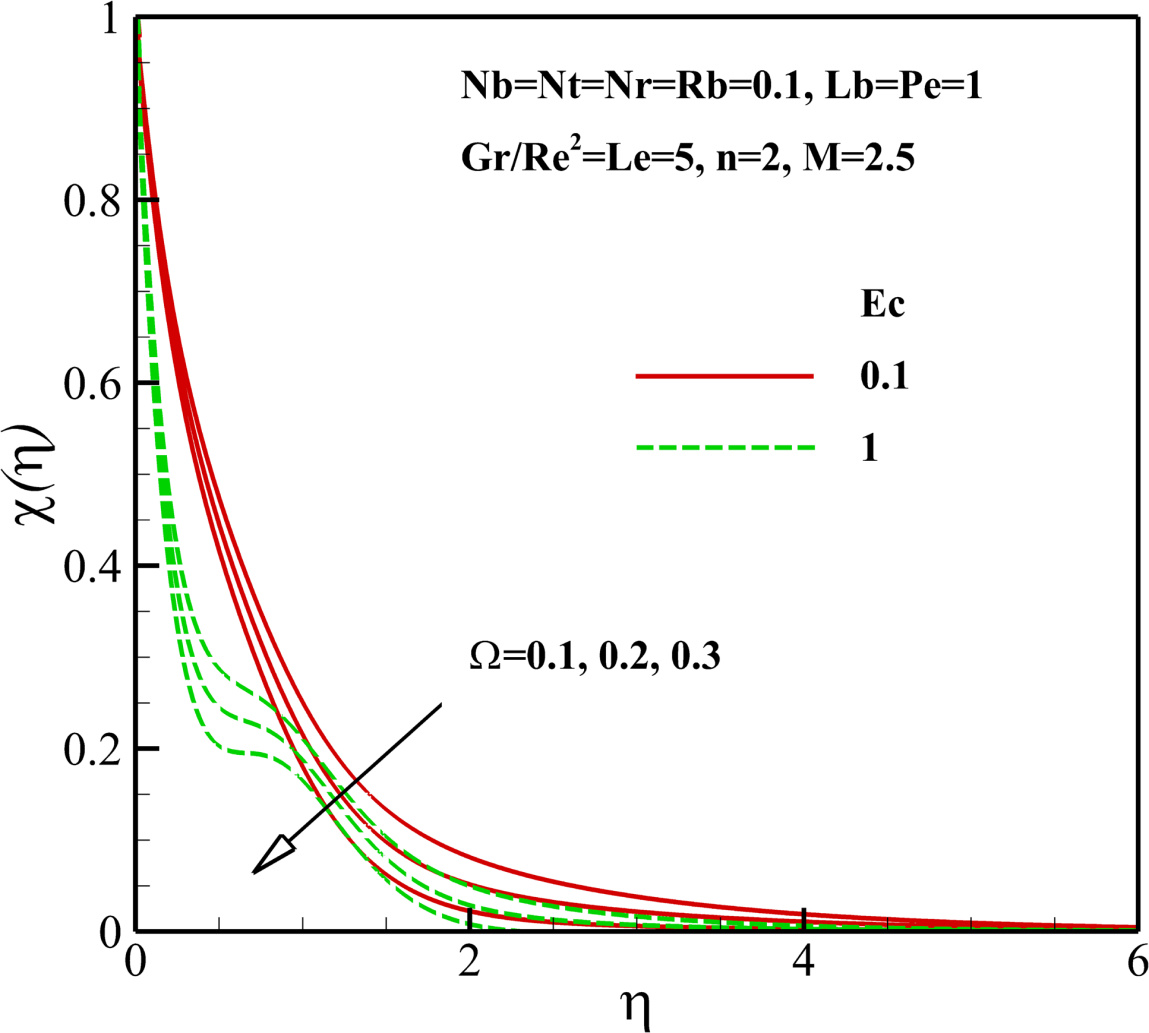
Effects of *Ec* and *Pe* on the density of motile microorganisms.

Figs 15–18 depict the variation of density of gyrotactic micro-organisms with coordinate *η* for different thermophysical parameters. Like the nanoparticles concentration, the presence of the magnetic field causes a decrease of the density of motile micro-organisms near the stretching sheet whereas increases the density motile micro-organisms away from it as is shown Fig 15. As already noted, the dimensionless velocity decreases with the increase of nonlinear stretching parameter *n*. Consequently, the boundary layer and density of motile micro-organisms boosts with an increasing in nonlinear stretching parameter n (see Fig 15). From Fig 16, it is observed that the density of motile micro-organisms decreases due to an increase in the dimensionless velocity with increasing Richardson number *Gr/Re*^*2*^. The increasing Richardson number *Gr/Re*^*2*^ also decreases the motile micro-organisms boundary layer thickness and as a result the motile micro-organisms flux increases with an increase in Richardson number *Gr/Re*^*2*^. As represented in Fig 17, the density of motile micro-organisms strongly decreases as bioconvection Lewis number *Lb* and Peclet number *Pe* increase. In fact, increasing in bioconvection Lewis number *Lb* and Peclet number *Pe* means the decrease of micro-organisms diffusion, so it is clear that both the density and boundary layer thickness for motile micro-organisms declines as growing of *Pe* and Lb. The reduction of both the density and boundary layer thickness for motile micro-organisms with an increase in Eckert number *Ec* and micro-organisms concentration difference parameter *Ω* is indicated in Fig 18.

### The Local Skin Friction *C*_*fx*_, Local Nusselt Number *Nu*_*x*_, Local Sherwood Number *Sh*_*x*_ and Local Density Number of the Motile Micro-Organisms *Nn*_*x*_

**Fig 19:**
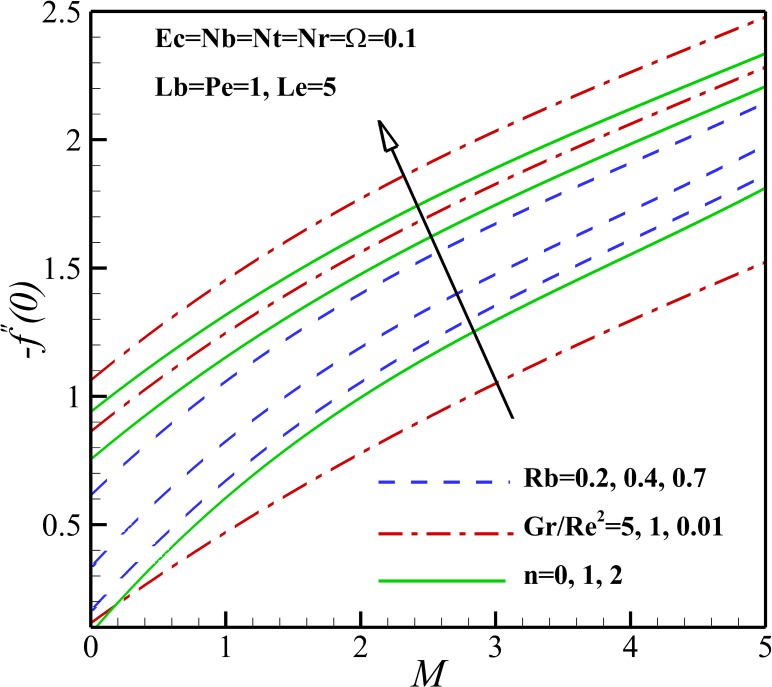
Effects of *M*, *n*, *Gr/Re*^*2*^ and *Rb* on the skin friction coefficient.

**Fig 20:**
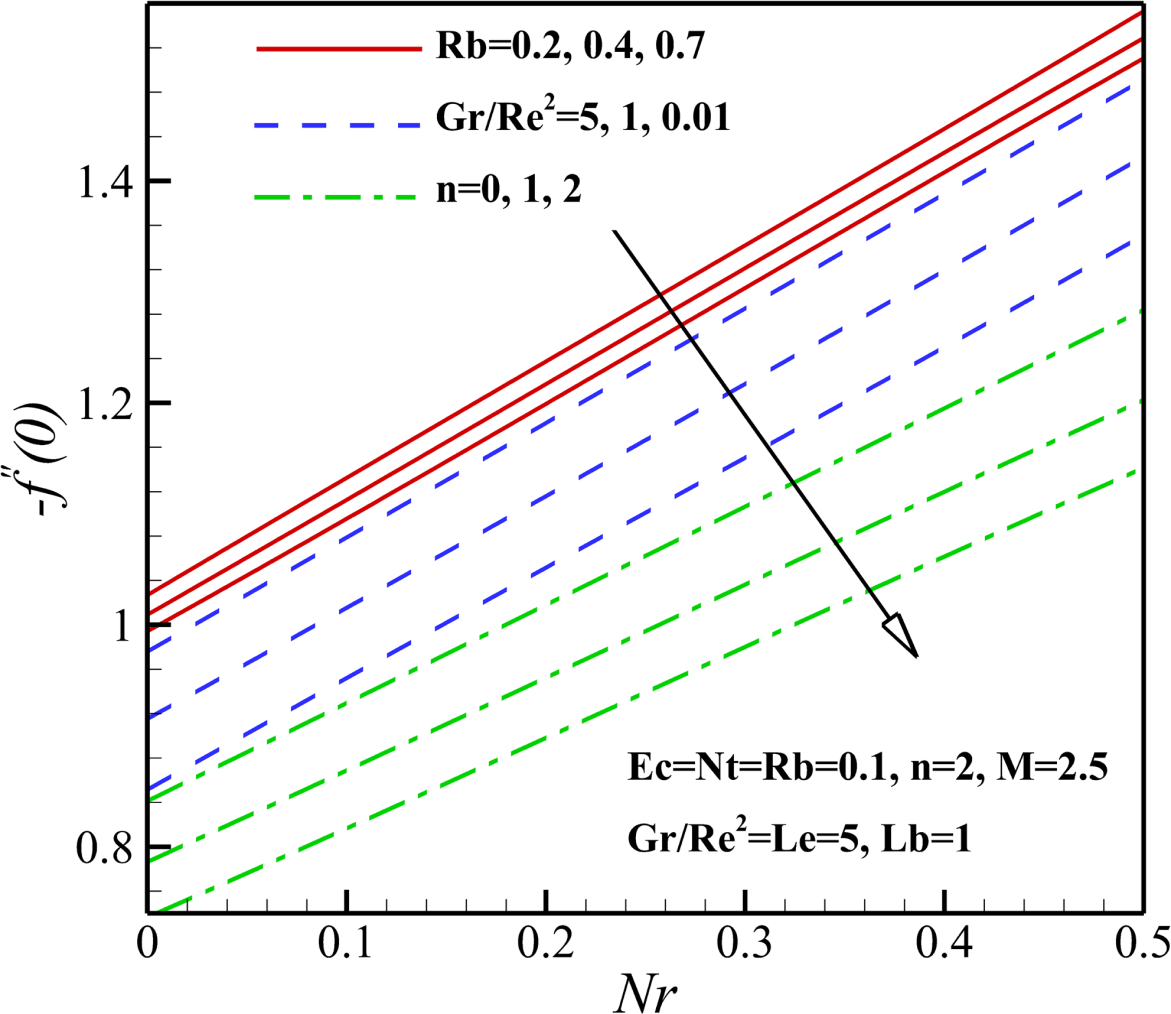
Effects of *Nr*, *Pe*, *Ω* and *Nb* on the skin friction coefficient.

Figs 19 and 20 illustrate the influence of the different thermophysical parameters on the skin friction coefficient *C*_*fx*_. Our results in Figs 19 and 20 state that the local skin friction coefficient *C*_*fx*_ amplifies with the increase of magnetic parameter *M*, nonlinear stretching parameter n, bioconvection Rayleigh number Rb and buoyancy ratio parameter *Nr*. This is due to the fact that the resistance of nanofluid containing of motile micro-organisms to flow increases with the increase of these parameters. Also, the different effects can be seen with the increase of Richardson number *Ri*, Peclet number *Pe*, Brownian motion parameter *Nb* and micro-organisms concentration difference parameter *Ω* as are shown in Figs 19 and 20.

**Fig 21:**
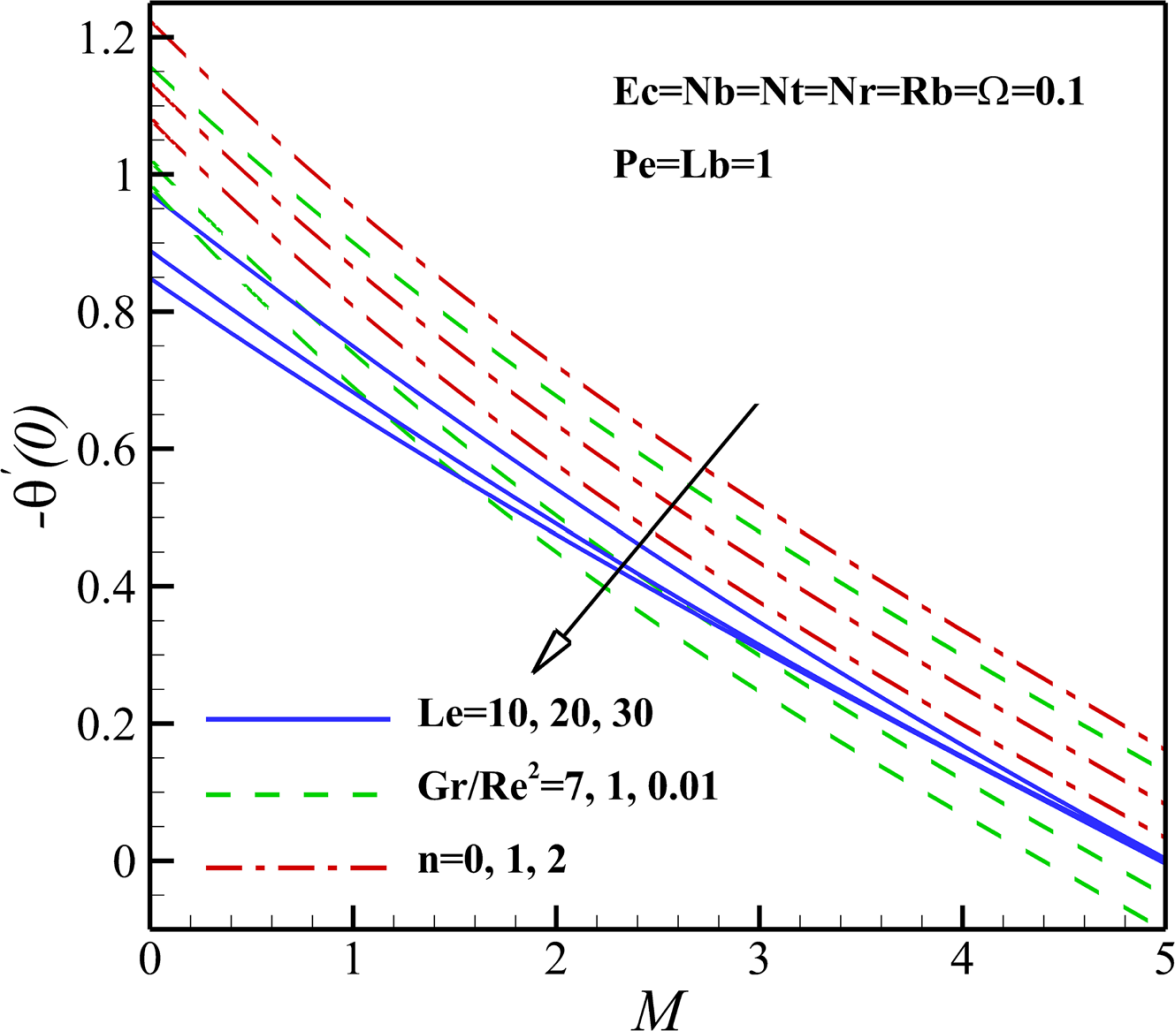
Effects of *M*, *n*, *Gr/Re*^*2*^ and *Le* on the Nusselt number.

**Fig 22:**
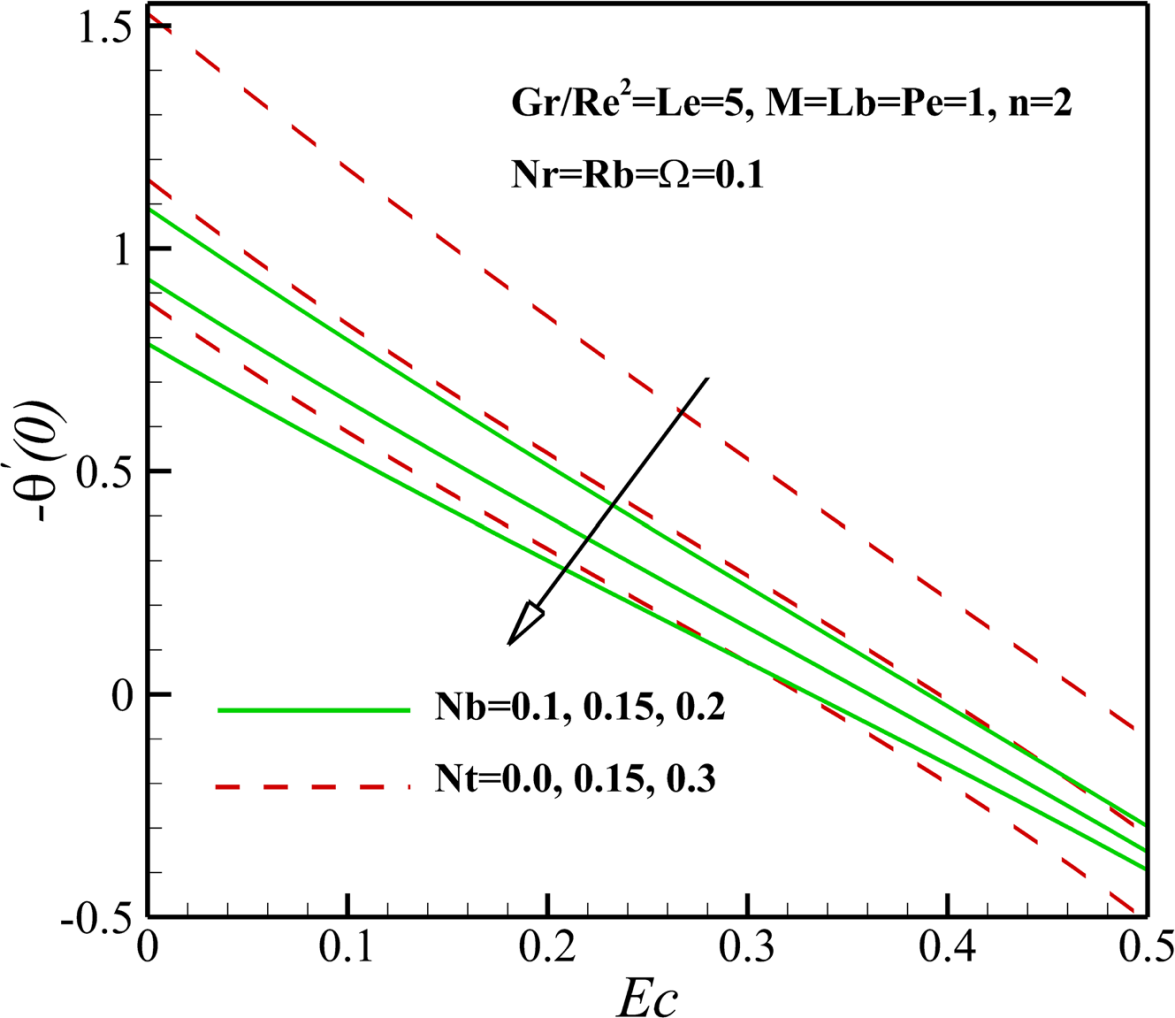
Effects of *Ec*, *Nb* and *Nt* on the Nusselt number.

**Fig 23:**
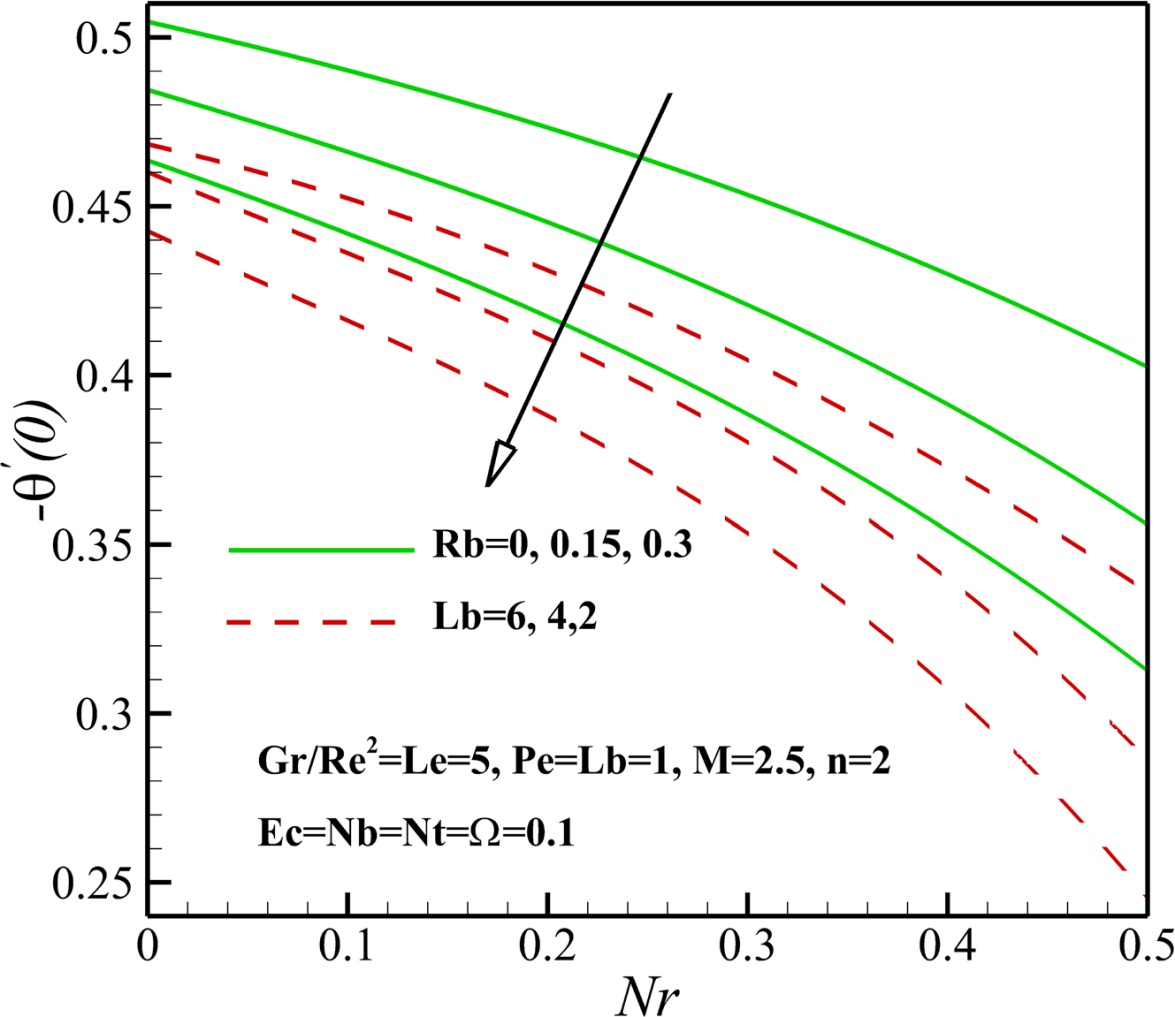
Effects of *Nr*, *Rb* and *Lb* on the Nusselt number.

The variation of local Nusselt number *Nu*_*x*_ with the different pertinent parameters is shown in Figs 21 and 22. Fig 23 demonstrates that the Nusselt number is decreasing with an increase in magnetic parameter *M*, nonlinear stretching sheet *n* and Lewis number *Le* and also with a decrease in *Gr/Re*^*2*^. This is due to the fact that the dimensionless temperature in the thermal boundary layer increases as magnetic parameter *M*, nonlinear stretching sheet *n* and Lewis number *Le* grows and Richardson number *Gr/Re*^*2*^ declines. As displayed in Fig 22, an increase in Eckert number *Ec*, Brownian parameter *Nb* and thermophoresis parameter *Nt* causes the decrease of the heat transfer rate from the stretching sheet. Same as before, the mentioned parameters enhances the dimensionless temperature in the thermal boundary layer and as a result the thermal boundary layer thickness enhances with the increase of Eckert number *Ec*, Brownian parameter *Nb* and thermophoresis parameter *Nt*. Also, Fig 23 shows that the heat transfer rate at surface increases with bioconvection Lewis number *Lb* and decreases with buoyancy ratio parameter *Nr* and the bioconvection Rayleigh number *Rb*. This can be attributed to the enhancement of dimensionless temperature of nanofluid containing gyrotactic micro-organisms due the increase of negative buoyancy with *Nr* and *Rb*.

**Fig 24:**
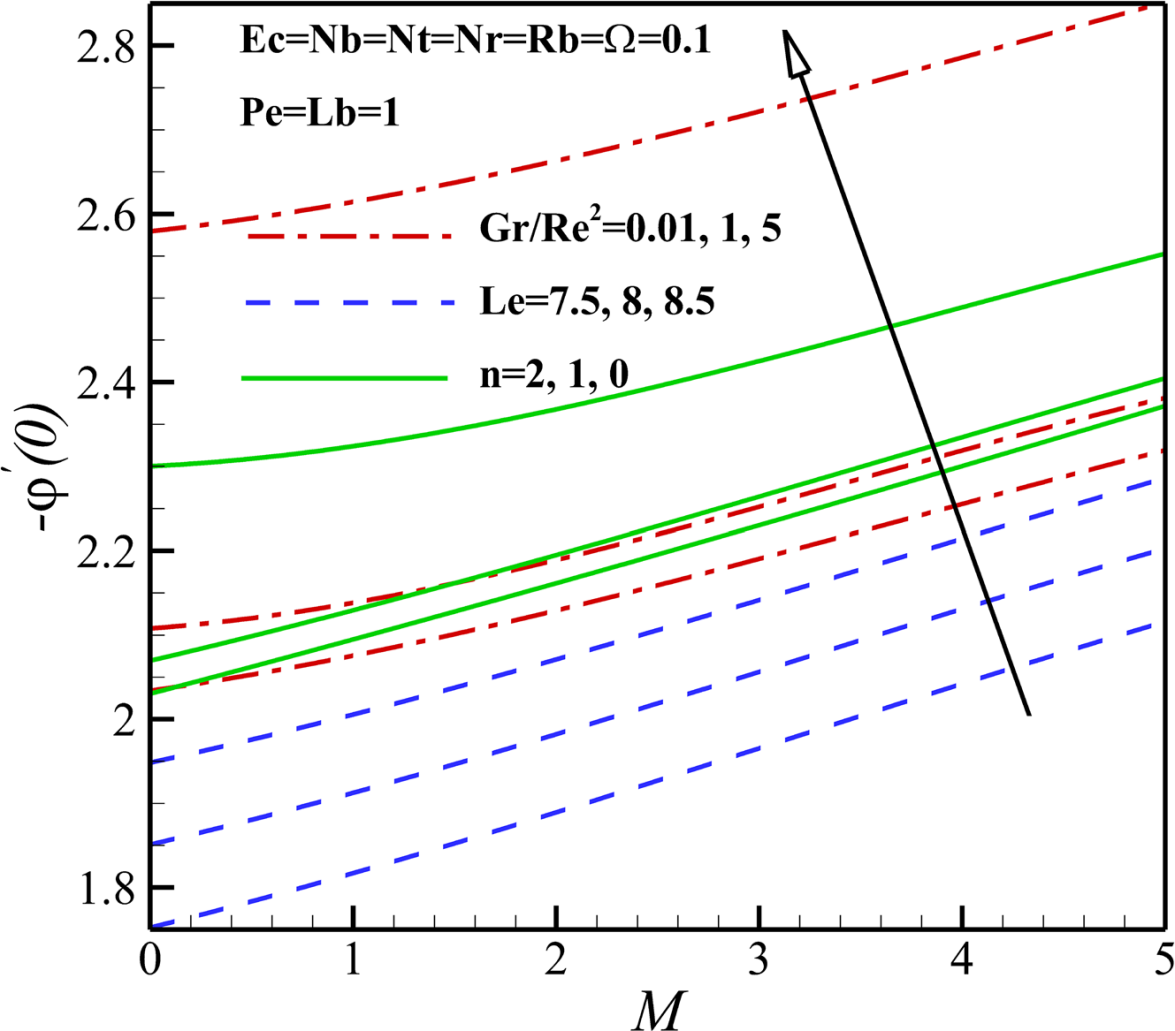
Effects of *M*, *n*, *Gr/Re*^*2*^ and *Le* on the Sherwood number.

As was shown in Fig 11, an increase in magnetic parameter *M* decreases the nanoparticles near the flat plate and this leads to a decline in the local Sherwood number *Sh*_*x*_ as represented in Fig 24. Due to increase in nonlinear stretching parameter *n*, the nanoparticles concentration increases, leading to a decline in the local Sherwood number *Sh*_*x*_. The mass transfer rate of the sheet or the local Sherwood number *Sh*_*x*_ also increases due to a decrease in the concentration of nanoparticles with increasing *Gr/Re*^*2*^ as shown in Fig 24. Finally, Fig 24 indicates that the local Sherwood number *Sh*_*x*_ rises enhances as Lewis number *Le* rises. This confirmed that the gradient of nanoparticles concentration profiles boosts with an increase in Lewis number *Le* as was introduced in Fig 12.

**Fig 25:**
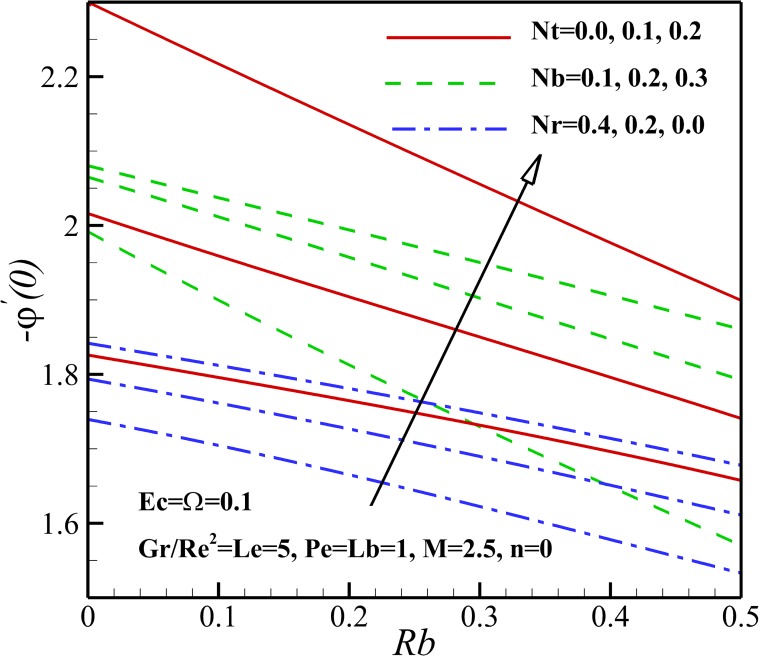
Effects of *Rb*, *Nt*, *Nb* and *Nr* on the Sherwood number.

Fig 25 depicts the variation of the local Nusselt number *Nu*_*x*_ on the stretching sheet with the variation of the thermophoresis parameter *Nt*, Brownian parameter *Nb*, the buoyancy ratio parameter *Nr* and the bioconvection Rayleigh number *Rb*. Our result shows that the rate of heat transfer increases with an increase of *Nb* and *Nt* due to the increase in the gradient of nanoparticles concentration profiles (see Figs 13 and 14). Also, it can be observed that Sherwood number rises with the increase of the buoyancy ratio parameter *Nr* and the bioconvection Rayleigh number *Rb*.

**Fig 26:**
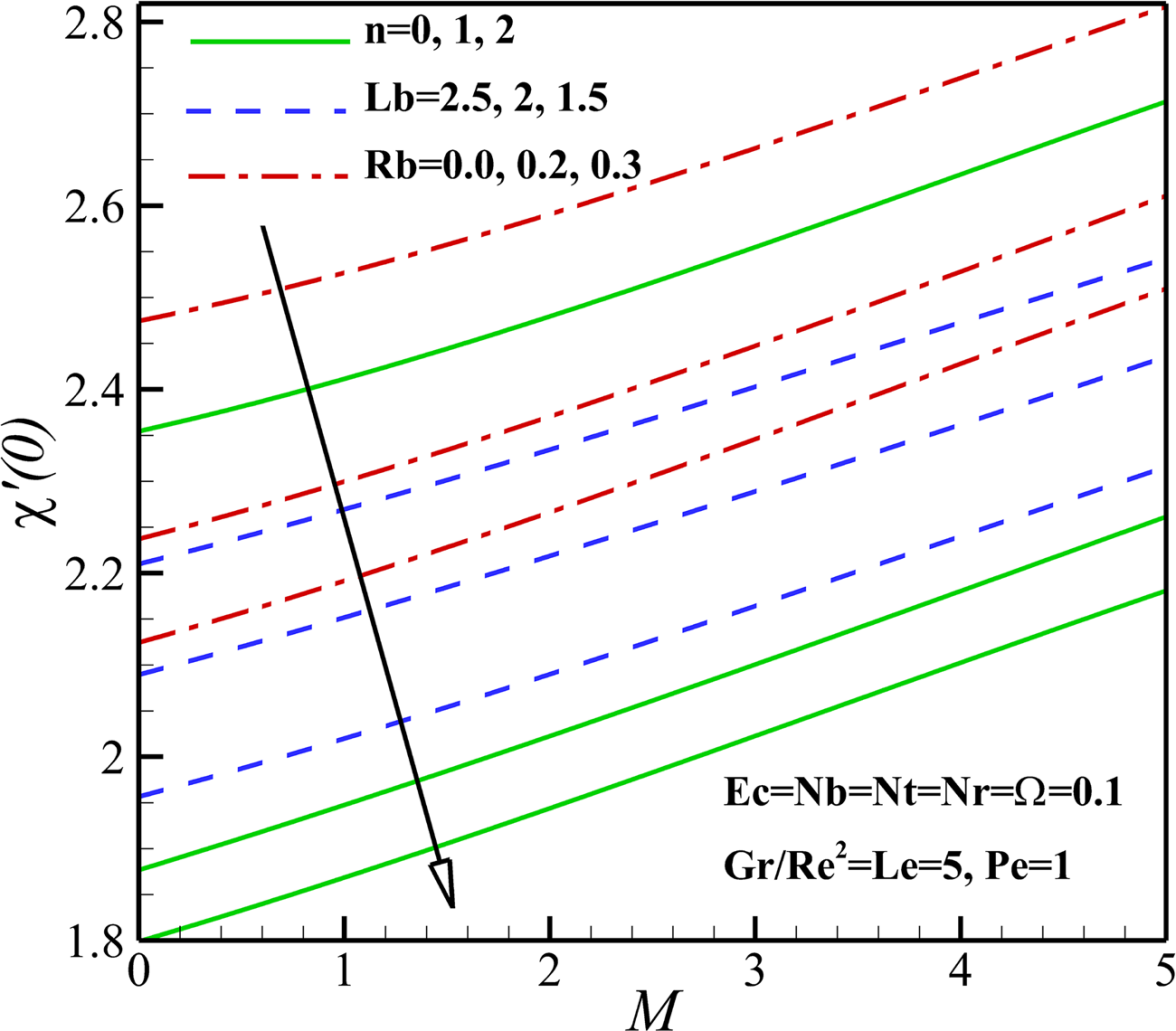
Effects of *M*, *n*, *Lb* and *Rb* on the density number of the motile micro-organisms.

**Fig 27:**
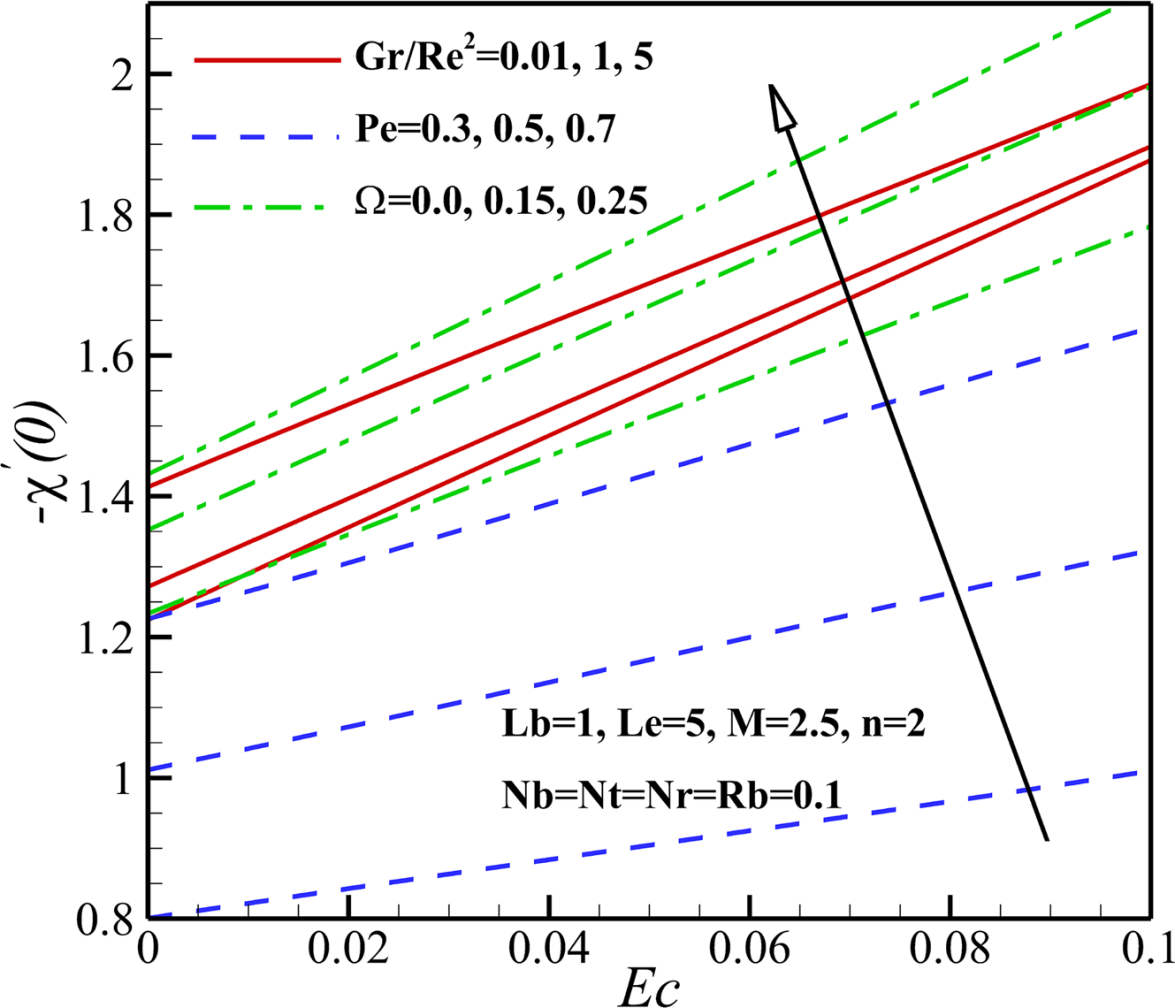
Effects of *Ec*, *Gr/Re*^*2*^, *Pe* and *Ω* on the density number of the motile micro-organisms.

The effect of magnetic parameter *M* on the density number of the motile micro-organisms for different *n*, *Rb* and *Lb* is depicted in Fig 26. It can be seen that the density number of the motile micro-organisms increases with an increase of magnetic parameter *M*. This is due to the reason that the magnetic field decreases the density of motile micro-organisms near the stretching sheet. With increasing of the nonlinear stretching parameter *n*, the density of motile micro-organisms augments within the boundary layer, leading to a reduction of the motile micro-organisms flux at the surface. The density number of the motile micro-organisms also decreases with increasing the bioconvection Rayleigh number *Rb* because of a reduction in the dimensionless velocity with increasing in the bioconvection Rayleigh number *Rb*. As bioconvection Lewis number *Lb* increases the density number of motile micro-organisms increase and this occurs since the convection of motile microorganism enhances with an increase of bioconvection Lewis number *Lb*. From Fig 27, an increase in the motile micro-organisms flux is noted with Eckert number *Ec*, Richardson number *Gr/Re*^*2*^, Peclet number *Pe* and the micro-organisms concentration difference parameter *Ω*. This would be attributed to the fact that the concentration of motile micro-organisms within the boundary layer for motile micro-organisms decreases as these parameters increase. These were shown in Figs 15–18.

## Conclusions

In the present paper, we have examined the boundary layer flow of a water-based nanofluid containing gyrotactic micro-organisms passing a nonlinear stretching vertical sheet in the presence of non-uniform magnetic field. The governing partial differential equations for mass, momentum, energy, concentration of nanoparticles, and motile micro-organisms density are converted into a system of the ordinary differential equations via a set of similarity transformations. These equations are numerically solved using an implicit finite difference method. The results of the investigation represent the following conclusions:

- The dimensionless temperature increases with the increase of bioconvection Rayleigh numbers *Rb* and buoyancy ratio parameter *Nr*. In contrast, it is seen that the temperature declines with Richardson number *Gr/Re*^*2*^, magnetic parameter *M* and nonlinear stretching parameter *n*.
- The nanoparticles concentration reduces near the stretching sheet and enhances away from it with an increase in magnetic parameter *M* and thermophoresis parameter *Nt*.
- The density of motile micro-organisms decreases as Richardson number *Gr/Re*^*2*^ and Eckert number *Ec* increase and increases with non-linear stretching parameter *n*. Like nanoparticles, the presence of magnetic field causes that the density of motile micro-organisms decreases in the vicinity of the sheet and increases far from it.
- The local skin fiction *C*_*fx*_ increases with the increase of magnetic parameter *M*, non-linear stretching parameter *n*, buoyancy ratio parameter *Nr*, bioconvection Rayleigh number *Rb*, whereas, decreases with Richardson number *Gr/Re*^*2*^, Peclet number *Pe*, Brownian motion parameter *Nb* and micro-organisms concentration difference parameter *Ω*.
- Increasing magnetic parameter *M*, non-linear stretching parameter *n*, Lewis number *Le*, Brownian motion parameter *Nb*, thermophoresis parameter *Nt*, bioconvection Rayleigh number *Rb*, buoyancy ratio parameter *Nr* and decreasing Richardson number *Gr/Re*^*2*^ and bioconvection Lewis number *Le* reduce the rate of heat transfer at the surface.
- Sherwood number rises with an increase in magnetic parameter *M*, Richardson number *Gr/Re*^*2*^, Lewis number *Le*, thermophoresis parameter *Nt*, however, diminishes with non-linear stretching *n*, bioconvection Rayleigh number *Rb*, Brownian motion parameter *Nb* and buoyancy ratio parameter *Nr*.
- The density number of the motile micro-organisms augments with magnetic parameter *M*, bioconvection Lewis number *Le*, Richardson number *Gr/Re*^*2*^, Peclet number *Pe* and *Ω* and reduces with bioconvection Rayleigh number *Rb* and non-linear stretching parameter *n*.
